# Selective enzymatic esterification of lignin model compounds in the ball mill

**DOI:** 10.3762/bjoc.13.173

**Published:** 2017-08-25

**Authors:** Ulla Weißbach, Saumya Dabral, Laure Konnert, Carsten Bolm, José G Hernández

**Affiliations:** 1 Institute of Organic Chemistry, RWTH Aachen University, Landoltweg 1, D-52074 Aachen, Germany

**Keywords:** ball milling, enzymes, esterification, lignin derivatization, mechanochemistry

## Abstract

A lipase-catalyzed esterification of lignin model compounds in the ball mill was developed combining the advantages of enzyme catalysis and mechanochemistry. Under the described conditions, the primary aliphatic hydroxy groups present in the substrates were selectively modified by the biocatalyst to afford monoesterified products. Amongst the tested lipases, CALB proved to be the most effective biocatalyst for these transformations. Noteworthy, various acyl donors of different chain lengths were tolerated under the mechanochemical conditions.

## Introduction

Mechanochemical reactions, particularly those carried out by ball milling, have recently attracted attention of a wider scientific community, owing to the many advantages the excellent mixing inside the ball mill can offer [1]. Besides avoiding or minimizing the use of organic solvents as reaction media, chemical transformations by ball milling very often take place more rapidly than their solution-based counterparts. Furthermore, mechanochemical reactions are known to afford products in higher yields with minimal formation of byproducts. In addition to this, mechanochemical activation has resulted in the discovery of otherwise inaccessible products or materials [2–3].

**Scheme 1:**
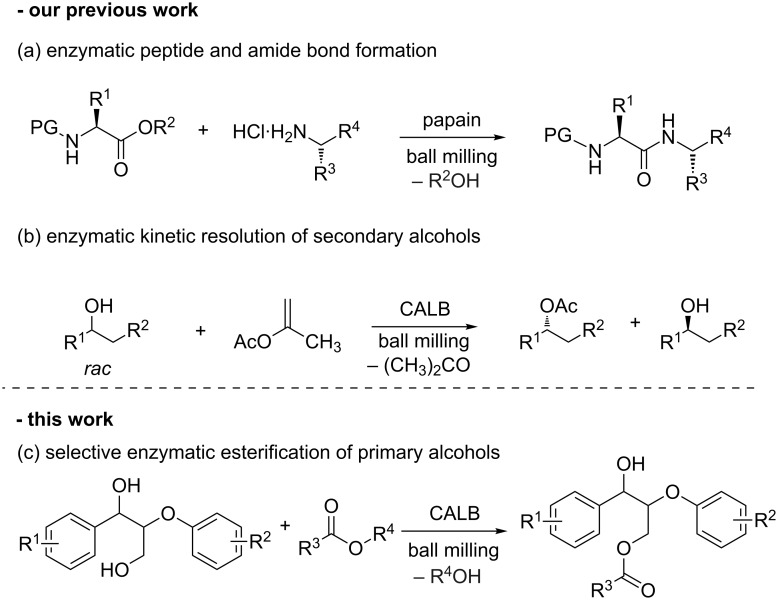
Enzymatic reactions under ball milling conditions.

In organic chemistry, amino acids and short peptides are not only known for being stable under automated ball milling conditions during their preparation [4], but also when applied as catalysts to perform stereoselective transformations [5–7]. Encouraged by these facts, we recently investigated the resilience of enzymes under ball milling conditions. The results from these studies have shown that biocatalysts such as cysteine and serine proteases tolerated the milling conditions and catalyzed the mechanoenzymatic peptide and amide bond formation after short milling times (Scheme 1a) [8].

Similarly, immobilized lipases (triglycerol acylhydrolases EC 3.1.1.3) such as Amano lipase PS-IM from *Burkholderia cepacia* immobilized on diatomaceous earth and lipase B from *Candida antarctica* (expressed *in Aspergillus niger*) adsorbed on polymethacrylate beads (ca. 400 μm–600 μm in diameter) [9], demonstrated to efficiently mediate the enzymatic kinetic resolution of secondary alcohols under solvent-free conditions in both mixer and planetary ball mills (Scheme 1b) [10]. Interestingly, this latter lipase (a commercial preparation known as Novozyme 345, hereinafter referred as CALB), showed the highest selectivity and could also be recycled by centrifugation and reused with little loss in stereoselectivity after four consecutive cycles [10].

Besides the above stated, one additional advantage of mechanochemistry includes the possibility to overcome solubility restrictions in chemical reactions involving reactants of poor solubility. This characteristic feature of mechanochemistry has proven fundamental while dealing with chemically induced cleavage of biomaterials such as lignin [11–12], cellulose [13–15] or chitin [16]. In regard to lignin chemistry, solution-based lignin depolymerization approaches or new applications of lignocellulose materials [17] often encounter solubility obstacles, forcing the alternate use of highly polar organic solvents, which thereby pose problems during metal-catalyzed transformations in the presence of strongly Lewis basic or donor solvents. In addition to this, miscibility and solubility of lignin samples in apolar matrices during the blending of lignin with polymeric materials is always a challenge.

To mitigate such solubility problems and to facilitate the utilization of lignin for various applications, efforts have been devoted to improve its lipophilicity, for instance through sulfation [18], silylation or esterification [19] of the aliphatic hydroxy and phenolic groups found in lignin. Chemical esterification of lignin [19–21] or its model compounds [22], using acetic anhydride in organic solvents such as DCM or pyridine have previously been reported to be effective in yielding new molecules and materials with higher hydrophobicity. However, controlling the degree of acetylation has not been an easy task, with the esterification process often resulting in a mixture of esters or fully esterified samples.

**Figure 1:**
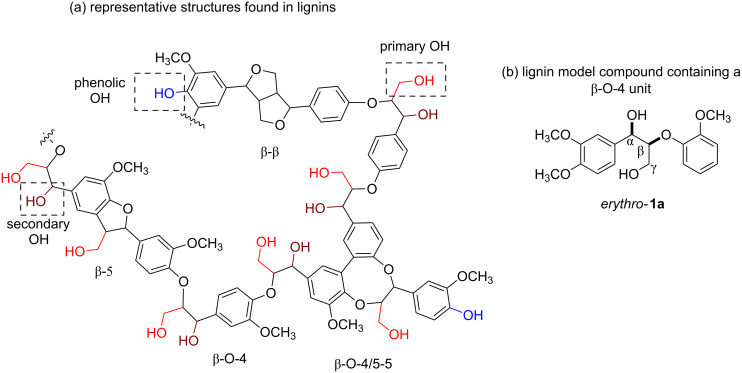
(a) Molecular representation of lignin. (b) Lignin model compound *erythro-***1a**.

In this regard, enzymatic esterification thus can be an attractive alternative to specifically address one type of hydroxy groups in the complex lignin structure. This could not only allow a selective control over the degree of hydrophilicity in lignin samples, but would also help tailoring their potential applications. One interesting approach in this field of study involves the modification of lignins by selectively esterifying the primary alcohols present in the biopolymer (Figure 1a), leaving untouched the phenolic and secondary alcohol functionalities, given that these functional groups have been associated with the biopolymer’s antioxidant, antibacterial and sun protection properties [17,23–24]. Motivated by the aforementioned scenario and in line with our research interest on studying the compatibility of biocatalysts and mechanochemical milling, we decided to investigate the enzymatic esterification of lignin model compounds in the ball mill (Scheme 1c). The results of this proof-of-concept study are presented here.

## Results and Discussion

**Scheme 2:**
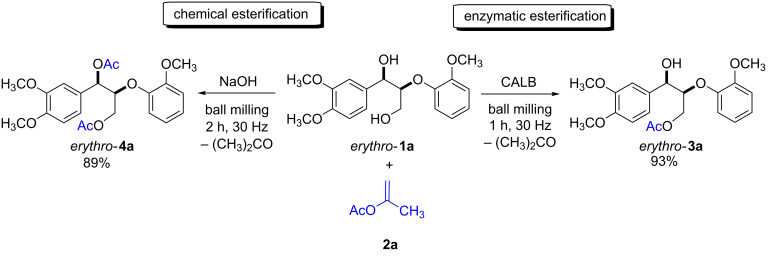
Chemical and enzymatic esterification of *erythro*-**1a** with isopropenyl acetate (**2a**) in the ball mill. Reaction conditions: *erythro*-**1a** (50 mg, 0.15 mmol), **2a** (0.60 mmol), CALB (30 mg of immobilized enzyme) or NaOH (12 mg, 0.30 mmol), 10 mL ZrO_2_ milling jar, 6 ZrO_2_ milling balls (5 mm in diameter).

Due to the high complexity of the lignin structure, which often presents a challenge during the product composition analysis, the use of lignin model compounds to monitor preliminary research advancements is a common practice [25–29]. Thus, for this investigation various dimeric compounds containing the β-O-4 linkage, primary and secondary hydroxy groups as well as several methoxy/phenolic moieties, were used. To begin with, we selected *erythro*-**1a** as a model compound to study the enzymatic esterification reactions in the ball mill (Scheme 2).

Based on our previous work [10], isopropenyl acetate (**2a**), a non-reversible acyl donor, was chosen as the acetylating agent. Milling a mixture of *erythro*-**1a** and **2a** for 2 h at 30 Hz did not afford any product, and only the reactants were observed by ^1^H NMR spectroscopy. Repeating the experiment in the presence of 30 mg of the immobilized lipase CALB led to a total conversion of the *erythro*-**1a** after just 1 h. Purification of the product by column chromatography afforded the monoacetylated *erythro*-**3a** in 93% yield (Scheme 2; right). To corroborate the role of the biocatalyst in the esterification, the experiment was repeated with sodium hydroxide in place of CALB. Consequently, after 2 h of milling the reaction only generated the diacetylated product *erythro*-**4a** (Scheme 2; left). These results reflect the high selectivity of the biocatalyst for primary hydroxy groups. In nature, lipases catalyze the hydrolysis of triglycerides, and are known for acting preferentially at the terminal position of triacylglycerol derivatives [30]. It is worth mentioning here that, even when *erythro*-**1a** was milled with an excess of acyl donor for longer time, CALB yielded exclusively the monoacetylated product *erythro*-**3a**.

Further screening of the reaction parameters revealed that lowering the amount of acyl donor was doable, although longer milling times were required. Similarly, the effect of the number of milling balls, frequency of milling, reaction time and additives was also investigated (Table S1 in Supporting Information File 1). In addition to this, the catalytic activity of a number of other lipases was studied (Table 1).

**Table 1:** Influence of various enzymes on the esterification of *erythro*-**1a** with isopropenyl acetate (**2a**) in the ball mill.^a^

Entry	Enzyme	**1a**:**3a** (%)^b^

1^c^	CALB	0:100
2	CALA	90:10
3	PS-IM	90:10
4	lipase A	100:0

^a^Reaction conditions: *erythro*-**1a** (50 mg, 0.15 mmol), enzyme (30 mg), **2a** (0.6 mmol), 10 mL ZrO_2_ milling jar, 6 ZrO_2_ milling balls (5 mm in diameter), milling time 2 h, milling frequency 30 Hz. ^b^Determined by ^1^H NMR spectroscopy. ^c^Milling time 1 h. CALB (lipase B from *Candida antarctica* (expressed in *Aspergillus niger*) adsorbed on polymethacrylate beads, known also as Novozyme 345); CALA (lipase A from *Candida antarctica,* immobilized on Immobead 150, recombinant from *Aspergillus oryzae*); PS-IM (Amano lipase from *Burkholderia cepacia* immobilized on diatomaceous earth); Lipase A (Amano lipase A from *Aspergillus niger*)*.*

Amongst the commercially available lipases, CALA (lipase A from *Candida antarctica*, immobilized on Immobead), immobilized lipase from *Burkholderia cepacia* (PS-IM) and lipase A from *Aspergillus niger* were tested. Firstly, hoping to find differences between the two hydrolases derived from *Candida antarctica*, an experiment using CALA was conducted. Despite CALB and CALA being produced by the same yeast, the latter proved less active at catalyzing the esterification of *erythro*-**1a** (Table 1, entry 2). This difference in reactivity between both of the lipases has been documented previously in the literature [31]. Comparably, lipase PS-IM, which has been reported to facilitate the acetylation of secondary β-nitro alcohols [32], and proved to be stable under ball milling conditions [10] exhibited lower catalytic activity than CALB (Table 1, entry 3). However, in both cases the alternative biocatalysts also afforded the monoacetylated dilignol derivative *erythro*-**3a**. Finally, lipase A showed no conversion of the substrate, which could be explained by its poor recognition of **1a** (Table 1, entry 4). Furthermore, a possible reason could be the reduced stability of the non-immobilized lipase when subjected to mechanochemical stress.

In the preliminary results, isopropenyl acetate (**2a**) proved highly efficient for the enzyme-catalyzed selective esterification of the model compound *erythro*-**1a**, partly due to the non-reversibility of the reaction. However, isopropenyl esters of carboxylic acids are, in general, not readily available. Therefore, in order to find alternative acyl donors for the biocatalyst in the ball mill, a series of acylating agents was screened (Table 2) [33].

**Table 2:** Screening of acyl donors for the selective monoacetylation of dilignol *erythro*-**1a**.^a^

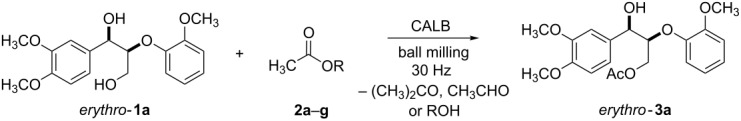			

Entry	R	Milling time (min)	**1a**:**3a** (%)^b^

1	isopropenyl (**2a**)	120	0:100
2	vinyl (**2b**)	120	6:94
3	phenyl (**2c**)	120	7:93
4	ethyl (**2d**)	120	70:30
5	isopropyl (**2e**)	120	66:34
6	allyl (**2f**)	120	63:37
7	*tert*-butyl (**2g**)	120	98:2
8^c^	H (**2h**)	90	100:0

^a^Reaction conditions: *erythro*-**1a** (50 mg, 0.15 mmol), CALB (30 mg of immobilized enzyme), acyl donor (0.60 mmol), 10 mL ZrO_2_ milling jar, 6 ZrO_2_ milling balls (5 mm in diameter). ^b^Determined by ^1^H NMR spectroscopy. ^c^10 equiv of **2h** were used.

Out of all the acyl donors tested, vinyl acetate (**2b**) and phenyl acetate (**2c**) were recognized and transferred by the lipase CALB to the acceptor *erythro*-**1a**, affording selectively the product *erythro*-**3a** (Table 2, entries 2 and 3). Notably, ethyl acetate (**2d**), isopropyl acetate (**2e**) and allyl acetate (**2f**) were suitable for the enzymatic esterification of *erythro*-**1a** as well, although to a lesser extent (Table 2, entries 4–6). Finally, lower and no reactivity was observed using *tert*-butyl acetate (**2g**) and acetic acid (**2h**) as acyl donor, respectively (Table 2, entries 7 and 8).

**Scheme 3:**
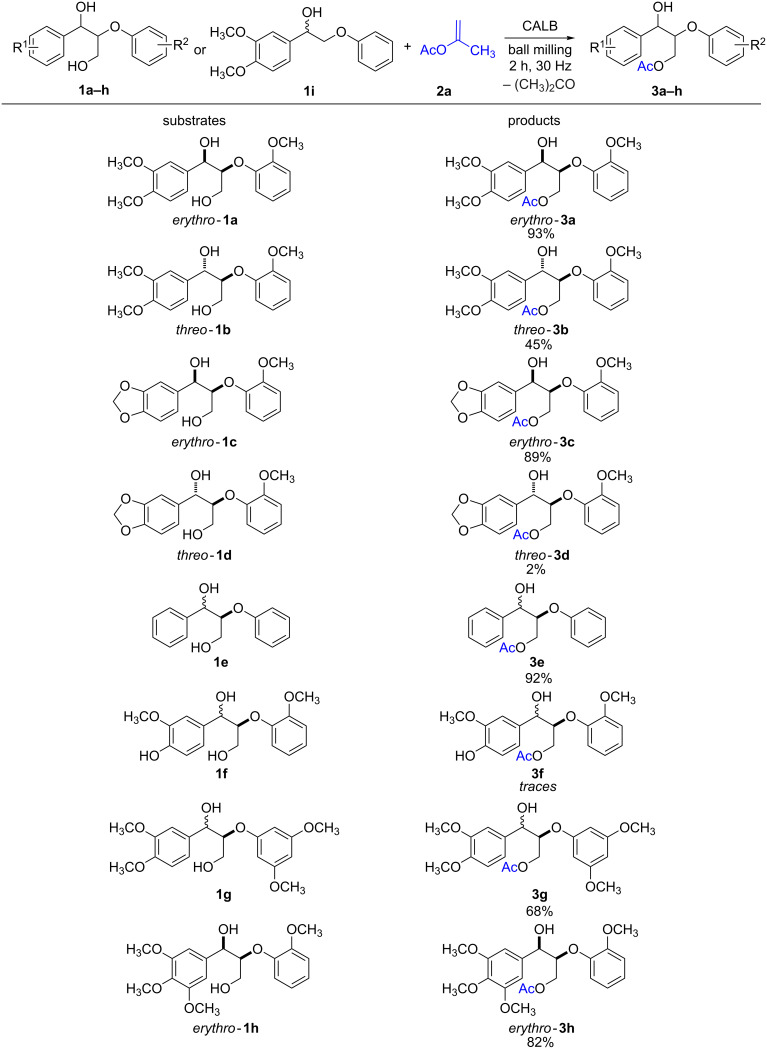
CALB-catalyzed esterification of lignin model compounds in the ball mill.

Having determined the best reaction conditions for the selective enzymatic acetylation of the *erythro*-**1a** in the ball mill, the protocol was applied to other β-O-4 model compounds (Scheme 3).

In general, all the substrates **1a**–**h** generated the monoacetylated derivatives, and the reactions occurred regioselectively at the primary hydroxy group of the model compounds. The regioselectivity of the reaction was further confirmed after the milling of isopropenyl acetate (**2a**) and the monolignol **1i**, only containing a benzylic alcohol. After the standard milling time, analysis of the reaction mixture by ^1^H NMR spectroscopy showed no product formation.

Moreover, under the standard reaction conditions, it was observed that the model compound *threo*-**1b** reacted slower in comparison to its diastereomer *erythro*-**1a**. After 2 h of milling, the product *threo*-**3b** was isolated in 45% yield (Scheme 3). These results highlight the importance of the stereochemistry of the substrates when interacting with the chiral biocatalyst. The reaction of the *erythro-*diastereoisomer **1c** showed comparable reactivity to *erythro*-**1a**, and the corresponding monoacetylated product *erythro*-**3c** could be isolated in 89% yield (Scheme 3). On the other hand, its diastereomer *threo*-**1d** was much less reactive and only trace quantities of *threo*-**3d** could be isolated. This difference in reactivity, which follows the trend previously observed for the pair *erythro*-**1a** and *threo*-**1b**, could have stemmed from matched/mismatched interactions of the diastereomeric diols and the chiral biocatalyst. Similarly, the unsubstituted model compound **1e** reacted smoothly to give **3e** in 92% yield. Purification of **3e** was done by filtration through a pad of celite, since it proved unstable towards standard purification procedures by column chromatography on silica gel.

Noteworthy is the low reactivity of the substrate **1f** bearing a phenolic group in its structure. In this case, only trace quantities of the monoacetylated product **3f** were observed after 2 h of milling and no esterification was seen to occur in the phenolic group. Initially, it was hypothesized that the presence of a phenolic functionality present in **1f** could have inhibited the lipase activity or perhaps caused some degree of denaturation in the enzyme. To test this hypothesis, control experiments using *erythro*-**1a**, **2a** and CALB in the presence of phenol (1.0 equiv) and phenol derivatives (guaiacol, 3-methoxyphenol, etc.) were carried out. In most cases, the presence of the additives had no negative effect on the performance of CALB (for details see Table S2 in Supporting Information File 1). Only the presence of 2,2’-biphenol seemed to have slowed down the acetylation of *erythro*-**1a**. A plausible explanation could be the nature of the 2,2’-biphenol moiety, which could have acted as a ligand interfering with the enzyme.

The resilience of CALB to phenols is in agreement with the high reactivity observed when phenyl acetate (**2c**), *erythro*-**1a** and CALB were milled (Table 2, entry 3), and formation of phenol was expected as a byproduct of the reaction. Hence, the lower reactivity of **1f** could have been a consequence of aggregation of the substrate or possible changes in its conformation. This could have reduced the affinity of CALB for **1f** compared to the non-phenolic counterparts. Additionally, milling experiments between **1f** and **2a**, where twice the amount of the enzyme was added in small portions, afforded the same negative result. Finally, the screening of the more hindered lignin model compounds **1g** and **1h** revealed that these substrates also reacted well in the ball mill, generating the monoacetylated derivatives **3g** and **3h** in 68% and 82% yield, respectively (Scheme 3).

**Scheme 4:**
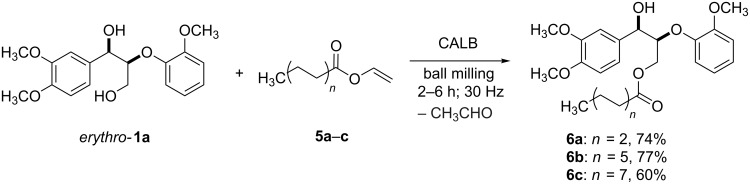
Selective esterification of *erythro*-**1a** using long-chain vinyl esters as acyl donors in the ball mill.

To test the catalytic efficiency of CALB in the ball mill, we decided to evaluate the performance of the biocatalyst in the esterification of *erythro*-**1a** using saturated fatty esters as acyl donors (Scheme 4).

Because of the excellent affinity of CALB for vinyl acetate (**2b**, Table 2, entry 2), and due to the commercial availability of vinyl esters in contrast to their isopropenyl analogues [33], vinyl esters **5a–c** were chosen. Pleasingly, under the optimized milling reaction conditions (2 h, 30 Hz), *erythro*-**1a** and **5a** afforded the monoacetylated dilignol derivative **6a** in 74% yield (Scheme 4). On the other hand, lengthening the carbon chain of the acyl donor (e.g., **5b** and **5c**) resulted in slowing down the reaction speed. However, an increase in the milling time from 2 h to 6 h proved beneficial and both the long-chain fatty ester dilignol derivatives **6b**,**c** were isolated in good yields (Scheme 4).

## Conclusion

In summary, the lipase-catalyzed esterification of lignin model compounds under mechanochemical conditions was investigated. Experimental parameters such as milling time, milling frequency, presence of additives and different acyl donors were studied in detail. Amongst the various biocatalysts tested, the lipase CALB proved superior in terms of catalytic activity and stability in the ball mill. The high catalytic activity of the enzyme facilitated the monoacetylation of β-O-4 lignin model compounds in good to high yields. Additionally, the biocatalyst exhibited higher preference for the aliphatic primary hydroxy group at the γ-position of the substrates. The enzymatic acetylation protocol was easily transferred to the esterification of the model substrate using long-chain fatty esters. This result is of high importance for introducing, in a controlled manner, various degrees of hydrophobicity to the substrates. This possibility is anticipated to be beneficial for future research initiatives employing lignin samples. Along these lines, it is important to comment on the lower reactivity towards the esterification of the substrate containing a phenolic substituent **1f**. Although it is known that lignin samples contain units bearing aromatic phenols, these phenolic fragments are mostly located at the terminal sides of the biopolymer. Therefore, enzymatically addressing the centrally-located primary aliphatic hydroxyl content of lignins is still highly possible. This strategy is expected to allow the preservation of the phenolic and benzylic alcohol contents in modified lignins, in order to keep the antibacterial and antioxidant activities of this biopolymer.

## Experimental

All reagents were obtained from commercial suppliers and used without further purification. All lignin model compounds were prepared following the reported procedures [25,34].

Analytical TLC was performed on silica gel plates, and the products were visualized by UV detection (wavelength 254 nm). Ball milling experiments were conducted using a Fritsch Mini-mill PULVERISETTE 23. NMR measurements were performed on Bruker AV 400 or AV 600 instruments. High-resolution mass spectra (HRMS) were measured using a Thermo Scientific LTQ Orbitrap XL with positive ion mode.

### Enzymatic acetylation of *erythro*-**1a** with CALB in the ball mill

A mixture of *erythro*-**1a** (50 mg, 0.15 mmol), acyl donor **2** (0.60 mmol) and CALB (30 mg of the immobilized enzyme) was milled for 2 h to 6 h at 30 Hz in a 10 mL ZrO_2_ milling jar loaded with 6 ZrO_2_ milling balls (5 mm in diameter). After the milling was stopped, the reaction mixture was recovered from the milling jar, supported on silica gel and the product was purified by silica column chromatography.

## Supporting Information

File 1 Experimental procedures, optimization tables, characterization data and NMR spectra.
