# Supplementary material for: Selective enzymatic esterification of lignin model compounds in the ball mill
Source: Beilstein J Org Chem. 2017 Aug 25;13:1788–95. doi: 10.3762/bjoc.13.173 (PMC5588541; doi:10.3762/bjoc.13.173)
Supplement: File 1 — Experimental procedures, optimization tables, characterization data and NMR spectra. [file Beilstein_J_Org_Chem-13-1788-s001.pdf]

Supporting Information

for

Selective enzymatic esterification of lignin model compounds in the ball mill

Ulla Weißbach, Saumya Dabral, Laure Konnert, Carsten Bolm* and José G. Hernández*

Address: Institute of Organic Chemistry, RWTH Aachen University, Landoltweg 1, D-52074 Aachen, Germany

Email: Carsten Bolm - Carsten.bolm@rwth-aachen.de; José G. Hernández - jose.hernandez@oc.rwth-aachen.de

*Corresponding author

Experimental procedures, optimization tables, characterization data and NMR spectra

Table of contents

1. Experimental **S2**
2. General procedure for the esterification of *erythro*-1a with CALB in the ball mill **S2**
3. Table S1: Optimization of the enzyme-catalyzed transesterification of the model compound *erythro*-1a with isopropenyl acetate (2a) **S3**
4. Table S2: Influence of phenolic additives on the selective enzyme-catalyzed monoacetylation of dilignol *erythro*-1a. **S4**
5. Characterization of the products 3, 4 and 6 **S4**
6. References **S9**
7. Selected NMR spectra **S9**

1. Experimental

All reagents and enzymes were obtained from commercial suppliers and used without further purification. All lignin model compounds were prepared following the reported procedures [1,2].

Analytical TLC was performed with silica gel plates, and the products were visualized by UV detection (wavelength 254 nm).

Ball milling experiments were conducted using a Fritsch Mini-mill PULVERISETTE 23, using zirconium oxide milling media.

NMR analysis was performed on Bruker AV 400 or AV 600 instruments. Samples were diluted in $CDCl_3$. Evaluation of the obtained spectra, namely integration of signal areas, peak selection as well as assignment of coupling constants, was performed using MestReNova software. Chemical shifts (δ) are given in ppm relative to the residual solvent peak ($CDCl_3$, δ = 7.26 ppm).

Mass spectra were acquired on a Finnigan SSQ7000 (EI, 70 eV) spectrometer.

High resolution mass spectra (HRMS) were measured using a Thermo Scientific LTQ Orbitrap XL with positive ion mode.

2. General procedure for the esterification of *erythro*-1a with CALB in the ball mill

A mixture of *erythro*-**1a** (50 mg, 0.15 mmol), acyl donor **2** (0.60 mmol) and CALB (30 mg) was milled for 2 h to 6 h at 30 Hz in 10 mL ZrO_2 milling jar loaded with 6 ZrO_2 milling balls (5 mm in diameter). After the milling was stopped, the reaction mixture was recovered from the milling jar, supported on silica gel and the product was purified by silica column chromatography.

Products **6a–c** were purified by preparative TLC using as eluent DCM/methanol (100:1).

3. Table S1: Optimization of the enzyme-catalyzed transesterification of the model compound *erythro*-**1a** with isopropenyl acetate (**2a**).[a]

Entry	2a (equiv)	Milling balls	Milling time (min)	SiO_2 (mg)	1:3 (%)[b]
1	2	6	120	-	15:85
2	4	6	120	-	0:100
3	4	6	60	-	0:100
4	4	6	45	-	0:100
5	4	6	30	-	21:88
6	2	10	120	-	29:71
7	2	3	120	-	12:88
8	2	6	45	70	91:9
9	2	6	45	40	84:16
10	2	6	45	20	76:24
11[c]	2	6	45	20	86:14

[a]Reaction conditions: model compound *erythro*-**1a** (50 mg, 0.15 mmol), CALB (30 mg), **2a**, 10 mL ZrO_2 jar, milling frequency 30 Hz, [b]determined by ^{1}H NMR spectroscopy, [c]milling frequency 50 Hz

4. Table S2: Influence of phenolic additives on the selective enzyme-catalyzed monoacetylation of dilignol *erythro*-**1a**.[a]

Entry	Additive	1:3 (%)[b]
1	none	47:53
2	guaiacol	0:100
3	phenol	15:85
4	3-methoxyphenol	19:81
5	3,5-dimethoxyphenol	16:84
6	2,2'-biphenol	52:48
7	2-phenylphenol	15:85

[a]Reaction conditions: model compound *erythro*-**1a** (50 mg, 0.15 mmol), CALB (30 mg), **2a** (32 µL, 0.3 mmol), additive (as indicated, 1 equiv), 10 mL ZrO_2 jar, 6 ZrO_2 milling balls (5 mm in diameter), milling time 1 h, milling frequency 30 Hz [b]determined by ^{1}H NMR spectroscopy

5. Characterization of the products 3, 4 and 6

(2*S*,3*R*)-3-(3,4-Dimethoxyphenyl)-3-hydroxy-2-(2-methoxyphenoxy)propyl acetate (*erythro*-3a): ^{1}H NMR (600 MHz, $CDCl_3$): δ = 7.07 (dt, *J* = 8.4 Hz, 1.2 Hz, 2H), 6.99 (d, *J* = 1.8 Hz, 1H), 6.94 (dt, *J* = 8.1 Hz, 1.8 Hz, 2H), 6.88 (dd, *J* = 8.4 Hz, 1.8 Hz, 1H), 6.83 (d, *J* = 7.8 Hz, 1H), 4.90 (d, *J* = 3.6 Hz, 1H), 4.46-4.43 (m, 1H), 4.38 (dd, *J* = 12.0 Hz, 7.8 Hz, 1H), 4.12 (dd, *J* = 11.4 Hz, 3 Hz, 1H), 3.89 (s, 3H), 3.88 (s, 3H), 3.87 (s, 3H), 2.01 (s, 3H) ppm. $^{13}C\{^1H\}$ NMR (151 MHz, $CDCl_3$): δ = 170.9, 151.6, 149.0, 148.4, 146.9, 131.4, 124.2, 121.5, 120.8, 118.5, 112.2, 110.9, 109.2,

84.5, 71.9, 62.8, 55.9 (3C), 20.9 ppm. HRMS (ESI, 70 eV): m/z calculated for $[M+Na]^+$: 399.14142, found: 399.14063.

(2*S*,3*S*)-3-(3,4-Dimethoxyphenyl)-3-hydroxy-2-(2-methoxyphenoxy)propyl acetate (*threo*-3b): ^{1}H NMR (400 MHz, $CDCl_3$): δ = 7.14 (dd, *J* = 8.0 Hz, 1.6 Hz, 1H), 7.06 (ddd, *J* = 8.4 Hz, 7.2 Hz, 1.6 Hz, 1H), 6.95-6.89 (m, 4H), 6.82 (d, *J* = 8.0 Hz, 1H), 4.86 (d, *J* = 8.4 Hz, 1H), 4.24 (dd, *J* = 10.8 Hz, 3.2 Hz, 1H), 4.23-4.19 (m, 2H), 3.90 (s, 3H), 3.86 (s, 3H), 3.86 (s, 3H), 2.03 (s, 3H) ppm. $^{13}C\{^1H\}$ NMR (101 MHz, $CDCl_3$): δ = 170.6, 150.8, 149.1, 149.0, 148.0, 131.7, 124.0, 121.4, 120.4, 119.7, 112.1, 111.0, 109.7, 86.1, 74.2, 63.3, 55.9 (2C), 55.8, 20.8 ppm. HRMS (ESI, 70 eV): m/z calculated for $[M+Na]^+$: 399.14142, found: 399.14124.

(2*S*,3*R*)-3-(Benzo[*d*][1,3]dioxol-5-yl)-3-hydroxy-2-(2-methoxyphenoxy)propyl acetate (*erythro*-3c): ^{1}H NMR (600 MHz, $CDCl_3$): δ = 7.05 (t, *J* = 6 Hz, 2H), 6.92 (t, *J* = 8.4 Hz, 3H), 6.80 (d, *J* = 8.4 Hz, 1H), 6.76 (d, *J* = 7.8 Hz, 1H), 5.93 (s, 2H), 4.86 (d, *J* = 3.6 Hz, 1H), 4.42-4.40 (m, 1H), 4.36 (dd, *J* = 11.4 Hz, 7.2 Hz, 1H), 4.11 (dd, *J* = 11.4 Hz, 3.0 Hz, 1H), 3.88 (s, 3H), 2.00 (s, 3H) ppm. $^{13}C\{^1H\}$ NMR (151 MHz, $CDCl_3$): δ = 170.9, 151.5, 147.7, 147.0, 146.9, 132.9, 124.2, 121.4, 120.8, 119.5, 112.2, 108.1, 106.9, 101.0, 84.3, 72.1, 62.8, 55.8, 20.8 ppm. HRMS (ESI, 70 eV): m/z calculated for $[M+Na]^+$: 383.11012, found: 383.11029.

(2*S*,3*S*)-3-(Benzo[*d*][1,3]dioxol-5-yl)-3-hydroxy-2-(2-methoxyphenoxy)propyl acetate (*threo*-3d): ^{1}H NMR (600 MHz, $CDCl_3$): δ = 7.13 (dd, *J* = 7.8 Hz, 1.2 Hz, 1H), 7.06 (dt, *J* = 7.8 Hz, 1.2 Hz, 1H), 6.95-6.90 (m, 2H), 6.89 (d, *J* = 1.8 Hz, 1H), 6.84 (dd, *J* = 7.8 Hz, 1.8 Hz, 1H), 6.76 (d, *J* = 7.8 Hz, 1H), 5.94 (s, 2H), 4.85 (d, *J* = 8.4, 1H), 4.26 (dd, *J* = 12.0 Hz, 3.0 Hz, 1H), 4.19-4.16 (m, 1H), 3.98 (dd, *J* = 12.0 Hz,

5.4 Hz, 1H), 3.91 (s, 3H), 2.05 (s, 3H) ppm. $^{13}C\{^1H\}$ NMR (151 MHz, $CDCl_3$): δ = 170.6, 150.9, 147.9, 147.8, 147.6, 133.1, 124.2, 121.4, 120.8, 120.6, 112.1, 108.3, 107.3, 101.1, 86.1, 74.2, 63.1, 55.8, 20.8 ppm. **MS (EI, 70 eV)** m/z (%) = 360 (6) [M^+], 283 (12), 150 (100), 121 (16), 93 (17).

3-Hydroxy-2-phenoxy-3-phenylpropyl acetate (3e): ^1H NMR (400 MHz, $CDCl_3$): δ = 7.43-7.22 (m, 6H), 7.00-6.85 (m, 4H), 5.02 (d, *J* = 4.8 Hz, 1H), 4.62 (ddd, *J* = 8.4 Hz, 4.8 Hz, 3.6 Hz, 1H), 4.43 (dd, *J* = 12.0 Hz, 4.8 Hz, 1H), 4.26 (dd, *J* = 12.0 Hz, 3.6 Hz, 1H), 1.91 (s, 3H) ppm. $^{13}C\{^1H\}$ NMR (101 MHz, $CDCl_3$): δ = 171.1, 157.9, 139.6, 129.6 (2C), 128.4 (2C), 127.9, 126.3 (2C), 121.9, 116.7 (2C), 80.3, 73.0, 62.4, 20.7 ppm. HRMS (ESI, 70 eV): m/z calculated for $[M+Na]^+$: 309.10973, found: 309.10977.

3-Hydroxy-3-(4-hydroxy-3-methoxyphenyl)-2-(2-methoxyphenoxy)propyl acetate (3f): ^1H NMR (400 MHz, $CDCl_3$): δ = 7.15 (dt, *J* = 8.0 Hz, 1.6 Hz, 1H), 7.07 (dt, *J* = 8.0, 1.6 Hz, 1H), 6.94 (dq, *J* = 4.8 Hz, 1.6 Hz, 2H), 6.91 (bs, 1H), 6.87 (bs, 2H), 5.60 (s, 1H), 4.85 (d, *J* = 7.6 Hz, 1H), 4.27-4.19 (m, 2H), 4.03 (dd, *J* = 12.0 Hz, 1.3 Hz, 1H), 3.92 (s, 3H), 3.88 (s, 3H), 2.04 (s, 3H) ppm.

2-(3,5-Dimethoxyphenoxy)-3-(3,4-dimethoxyphenyl)-3-hydroxypropyl acetate (3g): ^1H NMR (600 MHz, $CDCl_3$): δ = 6.95 (d, *J* = 2.4 Hz, 1H), 6.91 (dd, *J* = 7.8 Hz, 1.8 Hz, 1H), 6.83 (d, *J* = 8.4 Hz, 1H), 6.15 (d, *J* = 1.8 Hz, 2H), 6.11 (t, *J* = 1.8 Hz, 1H), 4.86 (d, *J* = 6.0 Hz, 1H), 4.50 (dd, *J* = 10.8 Hz, 5.4 Hz, 1H), 4.24 (dd, *J* = 12.0 Hz, 4.2 Hz, 1H), 4.07 (dd, *J* = 12.0 Hz, 5.4 Hz, 1H), 3.86 (s, 6H), 3.74 (s, 6H), 1.97 (s, 3H) ppm. $^{13}C\{^1H\}$ NMR (151 MHz, $CDCl_3$): δ = 170.7, 161.5 (2C), 159.9, 149.1, 149.0, 131.8, 119.3, 111.0, 109.7, 95.2 (2C), 94.1, 80.3, 73.4, 62.6, 55.9 (2C), 55.4

(2C), 20.7 ppm. HRMS (ESI, 70 eV): m/z calculated for $[M+Na]^+$: 429.15199, found: 429.15207.

(2*S*,3*R*)-3-Hydroxy-2-(2-methoxyphenoxy)-3-(3,4,5-trimethoxyphenyl)propyl acetate (*erythro*-3h): ^{1}H NMR (600 MHz, $CDCl_3$): δ = 7.07-7.03 (m, 2H), 6.93-6.91 (m, 2H), 6.60 (s, 2H), 4.87 (d, *J* = 4.2 Hz, 1H), 4.45-4.43 (m, 1H), 4.40 (dd, *J* = 12.0 Hz, 7.8 Hz, 1H), 4.14 (dd, *J* = 11.4 Hz, 3.0 Hz, 1H), 3.86 (s, 6H), 3.83 (s, 3H), 3.81 (s, 3H), 2.00 (s, 3H) ppm. ^{13}C{^{1}H} NMR (151 MHz, $CDCl_3$): δ = 171.0, 153.2, 151.5, 146.8, 134.6, 124.1, 121.4, 120.6, 112.2, 103.1, 84.1, 72.2, 72.1, 62.7, 60.8, 60.8, 56.1, 56.1, 50.8, 50.8, 20.8 ppm. HRMS (ESI, 70 eV): m/z calculated for $[M+Na]^+$: 429.15199, found: 429.15173.

(1*R*,2*S*)-1-(3,4-Dimethoxyphenyl)-2-(2-methoxyphenoxy)propane-1,3-diyl diacetate (*erythro*-4a): ^{1}H NMR (600 MHz, $CDCl_3$): δ = 7.01 (d, *J* = 1.8 Hz, 1H), 6.98-6.95 (m, 2H), 6.87-6.80 (m, 4H), 6.01 (d, *J* = 5.4 Hz, 1H), 4.70-4.67 (m, 1H), 4.41 (dd, *J* = 11.4 Hz, 6.0 Hz, 1H), 4.22 (dd, *J* = 12.0 Hz, 4.2 Hz, 1H), 3.86 (s, 3H), 3.85 (s, 3H), 3.78 (s, 3H), 2.06 (s, 3H), 2.02 (s, 3H) ppm. ^{13}C{^{1}H} NMR (151 MHz, $CDCl_3$): δ = 170.8, 169.7, 151.0, 149.0, 148.8, 147.3, 128.9, 123.4, 120.9, 120.0, 119.2, 112.5, 110.8 (2C), 80.1, 74.1, 62.8, 55.9 (2C), 55.7, 21.1, 20.8 ppm. HRMS (ESI, 70 eV): m/z calculated for $[M+Na]^+$: 441.15199, found: 441.15216.

(2*S*,3*R*)-3-(3,4-Dimethoxyphenyl)-3-hydroxy-2-(2-methoxyphenoxy)propyl hexanoate (*erythro*-6a): ^{1}H NMR (400 MHz, $CDCl_3$): δ = 7.06-7.02 (m, 2H), 6.98 (d, *J* = 1.6 Hz, 1H), 6.92 (s, 1H), 6.90 (s, 1H), 6.87 (dt, *J* = 8.4, 1.6, 1H), 6.81 (d, *J* = 8.0 Hz, 1H), 4.87 (d, *J* = 3.6 Hz, 1H), 4.43 (dt, *J* = 7.6 Hz, 3.6 Hz, 1H), 4.36 (dd, *J* = 11.6 Hz, 7.2 Hz, 1H), 4.01 (dd, *J* = 12 Hz, 3.6 Hz, 1H), 3.86 (s, 1H), 3.84 (s, 1H), 3.84 (s,

1H), 2.22 (t, *J* = 7.2 Hz, 2H), 1.54 (p, *J* = 7.6 Hz, 2H), 1.28-1.21 (m, 4H), 0.84 (t, *J* = 6.8 Hz, 3H) ppm. $^{13}C\{^{1}H\}$ NMR (151 MHz, $CDCl_3$): δ = 173.7, 151.5, 149.0, 148.5, 147.0, 131.5, 124.1, 121.4, 120.6, 118.5, 112.2, 110.9, 109.4, 84.3, 72.1, 62.6, 55.9 (2C), 55.8, 34.1, 31.3, 24.5, 22.3, 13.9 ppm. HRMS (ESI, 70 eV): m/z calculated for $[M]^+$: 432.21426, found: 432.21495.

(2*S*,3*R*)-3-(3,4-Dimethoxyphenyl)-3-hydroxy-2-(2-methoxyphenoxy)propyl dodecanoate (*erythro*-6b): ^{1}H NMR (400 MHz, $CDCl_3$): δ = 7.06 (dt, *J* = 7.8 Hz, 1.2 Hz, 2H), 7.00 (d, *J* = 1.8 Hz, 1H), 6.95-6.92 (m, 2H), 6.88 (dd, *J* = 8.4 Hz, 2.4 Hz, 1H), 6.83 (d, *J* = 8.4 Hz, 1H), 4.89 (d, *J* = 4.2 Hz, 1H), 4.45 (dt, *J* = 7.2 Hz, 3.6 Hz, 1H), 4.38 (dd, *J* = 12.0 Hz, 7.8 Hz, H), 4.11 (dd, *J* = 11.4 Hz, 3.6 Hz, 1H), 3.89 (s, 3H), 3.88 (s, 3H), 3.87 (s, 3H), 2.25 (t, *J* = 7.8 Hz, 2H), 1.56 (q, *J* = 7.2 Hz, 2H), 1.31-1.23 (m, 16H), 0.88 (t, *J* = 7.2 Hz, 3H) ppm. $^{13}C\{^{1}H\}$ NMR (151 MHz, $CDCl_3$): δ = 173.7, 151.6, 149.0, 148.5, 147.0, 131.5, 124.1, 121.4, 120.8, 118.5, 112.2, 110.9, 109.4, 84.4, 72.0, 62.6, 55.9, 55.8, 34.2, 31.9, 29.6 (3C), 29.4, 29.3 (2C), 29.1, 24.8, 22.7, 14.1 ppm. HRMS (ESI, 70 eV): m/z calculated for $[M]^+$: 516.30816, found: 516.30781.

(2*S*,3*R*)-3-(3,4-Dimethoxyphenyl)-3-hydroxy-2-(2-methoxyphenoxy)propyl hexadecanoate (*erythro*-6c): ^{1}H NMR (400 MHz, $CDCl_3$): δ = 7.06-7.01 (m, 2H), 6.98 (d, *J* = 2.0 Hz, 1H), 6.91 (d, *J* = 7.6 Hz, 2H), 6.87 (dt, *J* = 8 Hz, 1.6 Hz, 2H), 6.80 (d, *J* = 8.0 Hz, 1H), 4.87 (t, *J* = 4.0 Hz, 1H), 4.43 (dt, *J* = 8.0 Hz, 4 Hz, 1H), 4.36 (dd, *J* = 12.0 Hz, 4.8 Hz, H), 4.09 (dd, *J* = 11.6 Hz, 4.8 Hz, 1H), 3.86 (s, 3H), 3.86 (s, 3H), 3.84 (s, 3H), 2.23 (t, *J* = 7.6 Hz, 2H), 1.54 (p, *J* = 7.2 Hz, 2H), 1.29-1.22 (m, 24H), 0.86 (t, *J* = 6.4 Hz, 3H) ppm. $^{13}C\{^{1}H\}$ NMR (101 MHz, $CDCl_3$): δ = 173.7, 151.5, 149.0, 148.4, 147.0, 131.5, 124.0, 121.4, 120.7, 118.5, 112.2, 110.9, 109.3, 84.4,

72.0, 62.6, 55.8 (2C), 34.1, 31.9, 29.7 (2C), 29.6 (5C), 29.4, 29.3, 29.2, 29.1, 24.8, 22.7, 14.1 ppm. HRMS (ESI, 70 eV): m/z calculated for $[M+Na]^{+}$: 595.36053, found: 595.35889.

6. References

1. Rahimi, A.; Azarpira, A.; Kim, H.; Ralph, J.; Stahl, S. S. *J. Am. Chem. Soc*. **2013**, *135*, 6415−6418. doi: 10.1021/ja401793n

2. Buendia, J.; Mottweiler, J.; Bolm, C. *Chem. Eur. J.* **2011**, *17*, 13877–13882. doi: 10.1002/chem.201101579

7. Selected NMR spectra

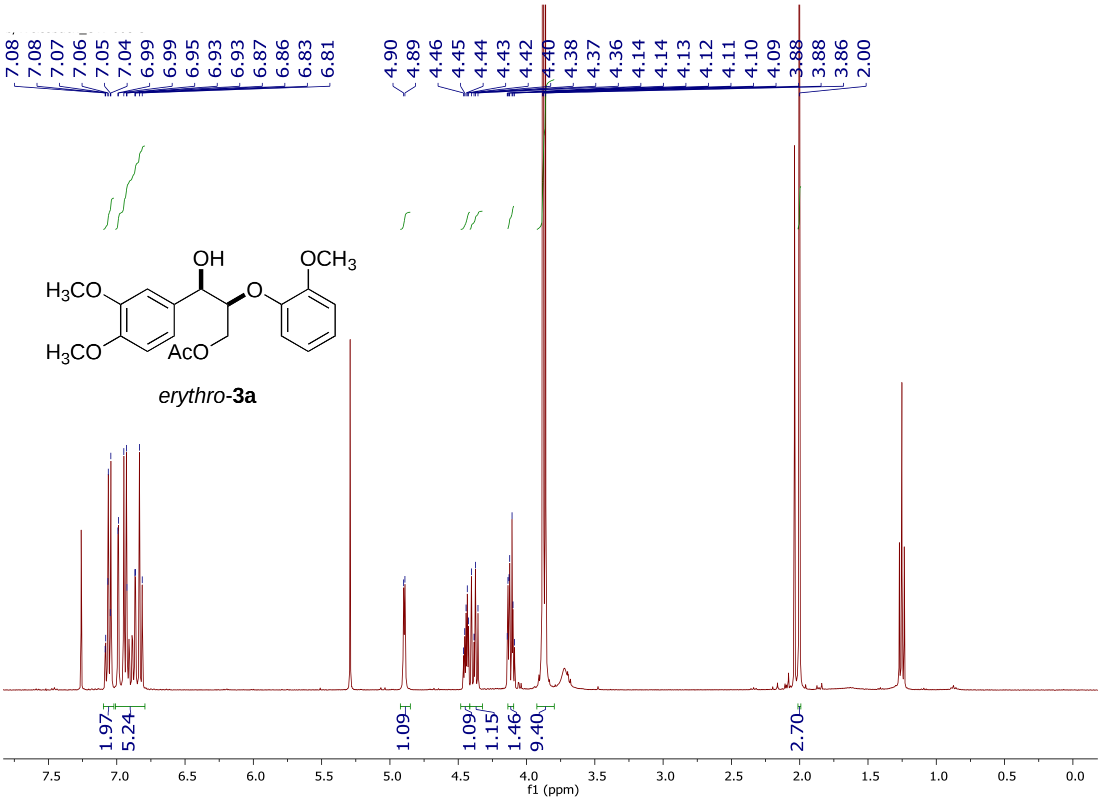

Figure S1: ^{1}H NMR spectrum of *erythro*-**3a.**

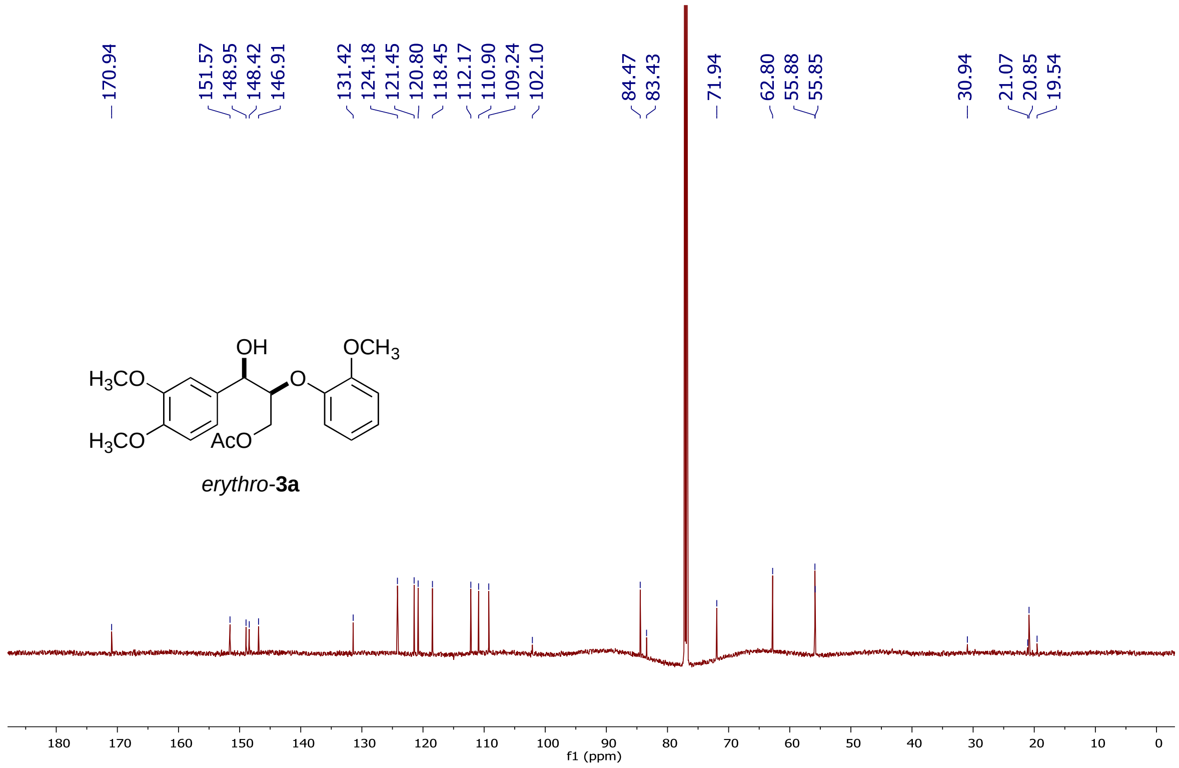

Figure S2: ^{13}C NMR spectrum of *erythro*-**3a.**

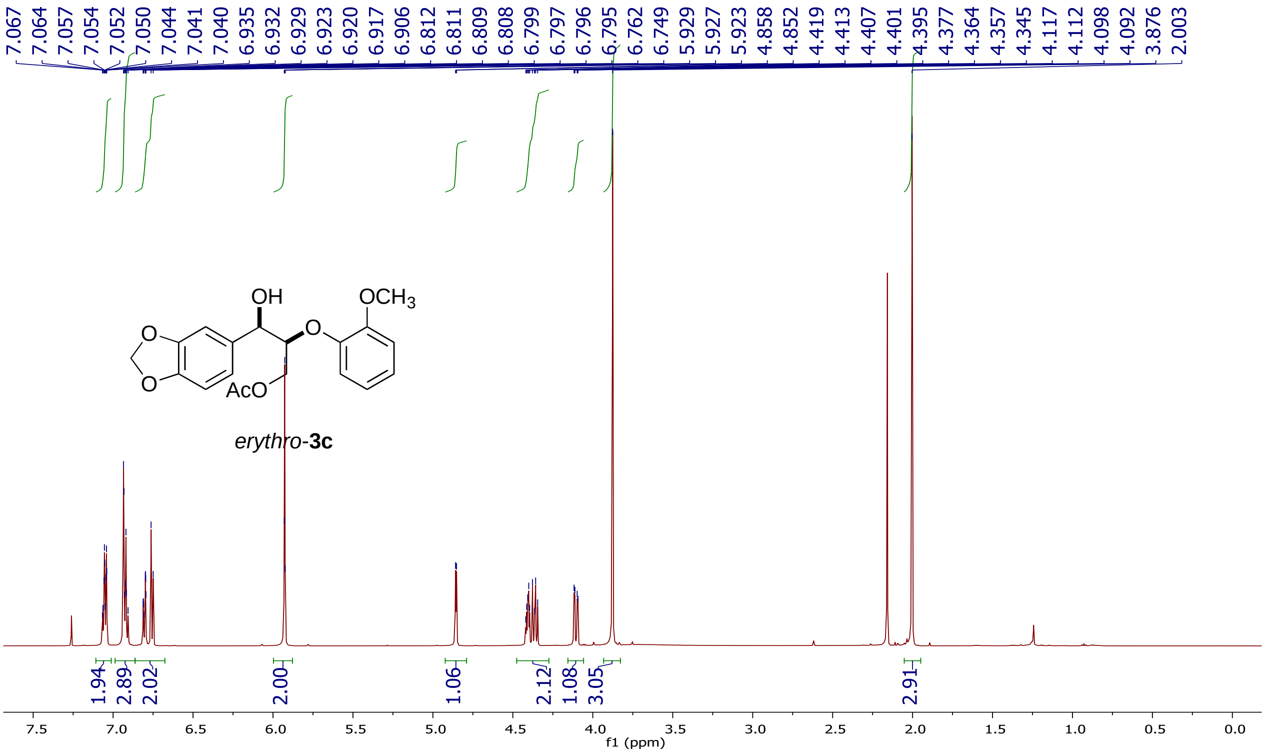

Figure S3: ^{1}H NMR spectrum of *erythro*-**3c.**

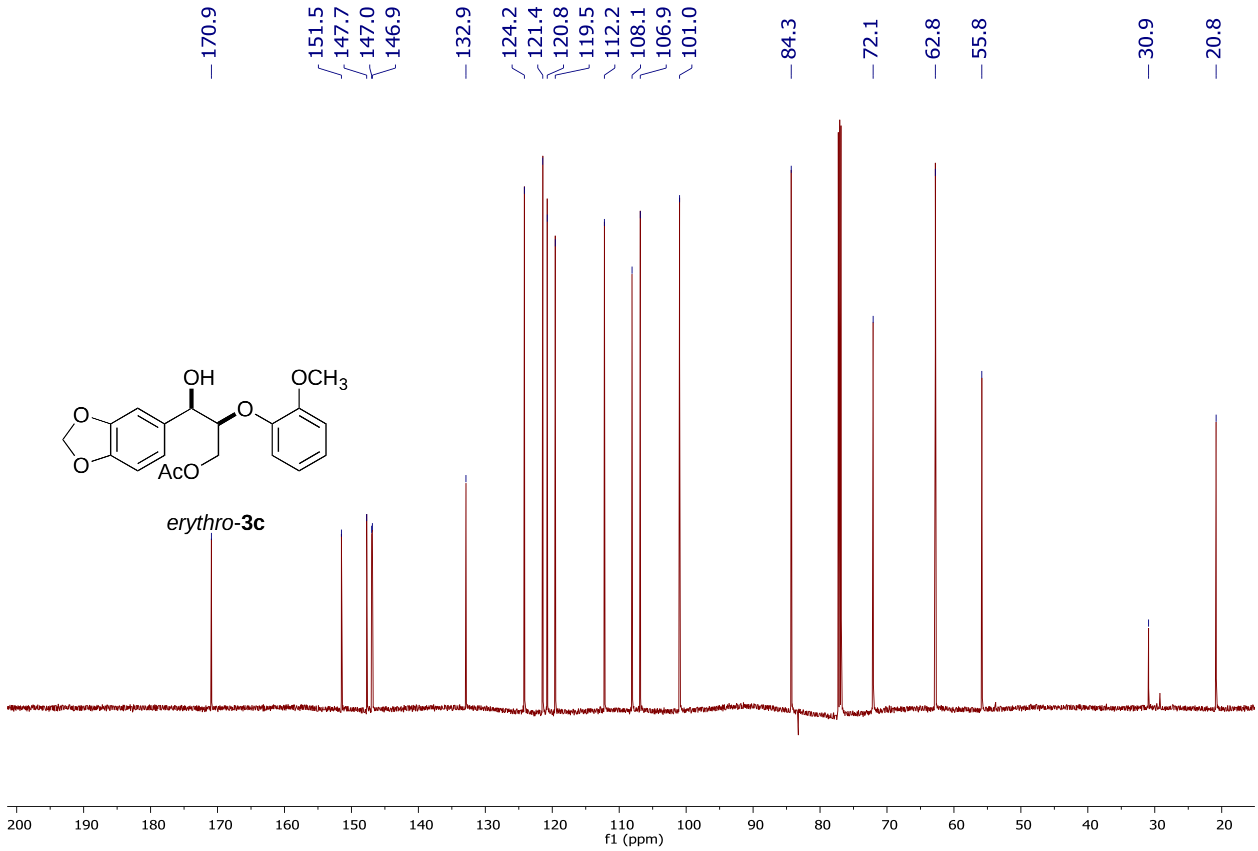

Figure S4: ^{13}C NMR spectrum of *erythro*-**3c.**

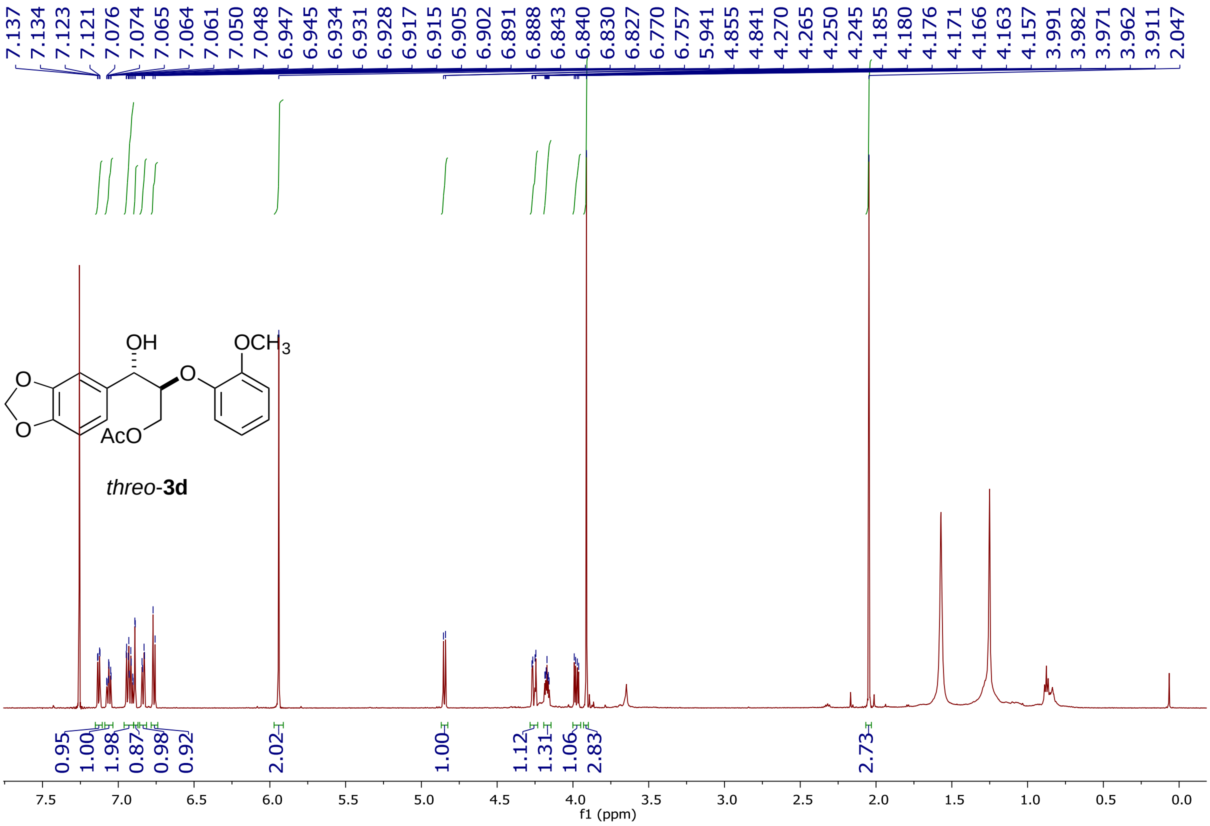

Figure S5: ^{1}H NMR spectrum of *threo*-**3d.**

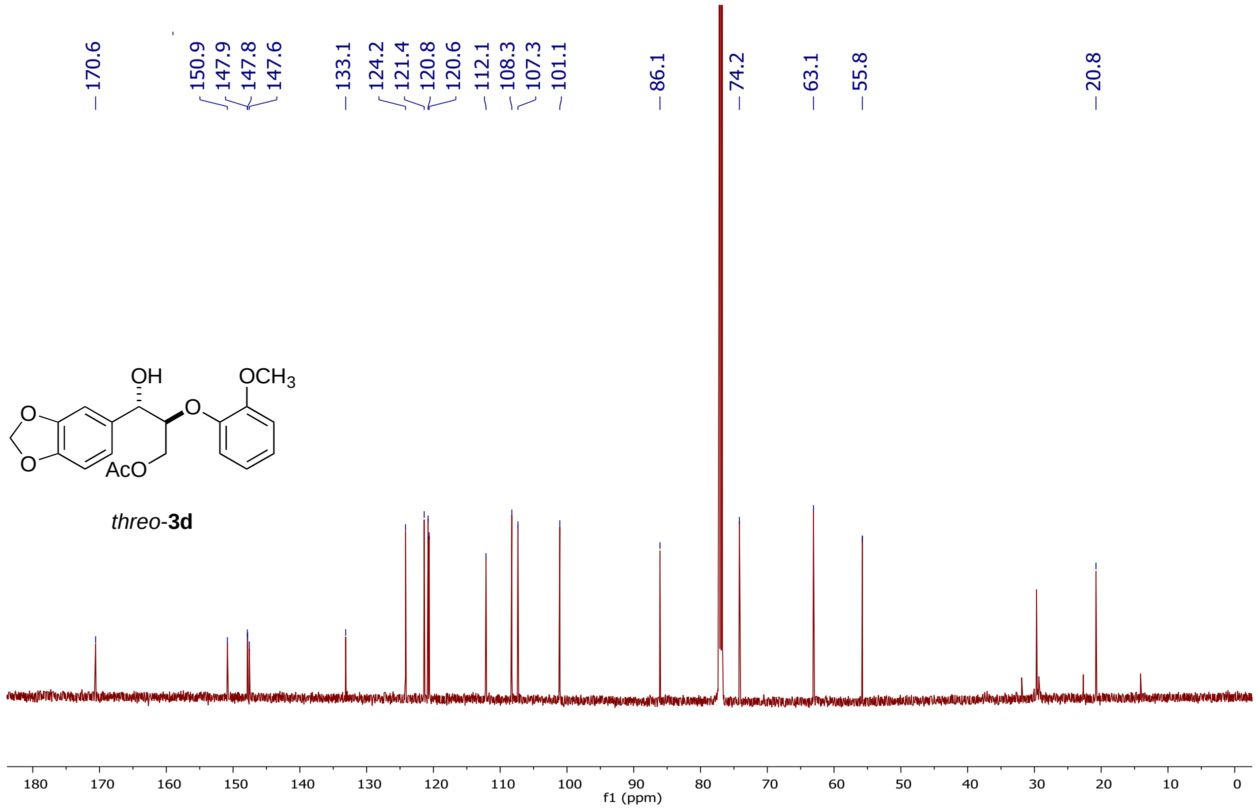

Figure S6: ^{1}H NMR spectrum of *threo*-**3d.**

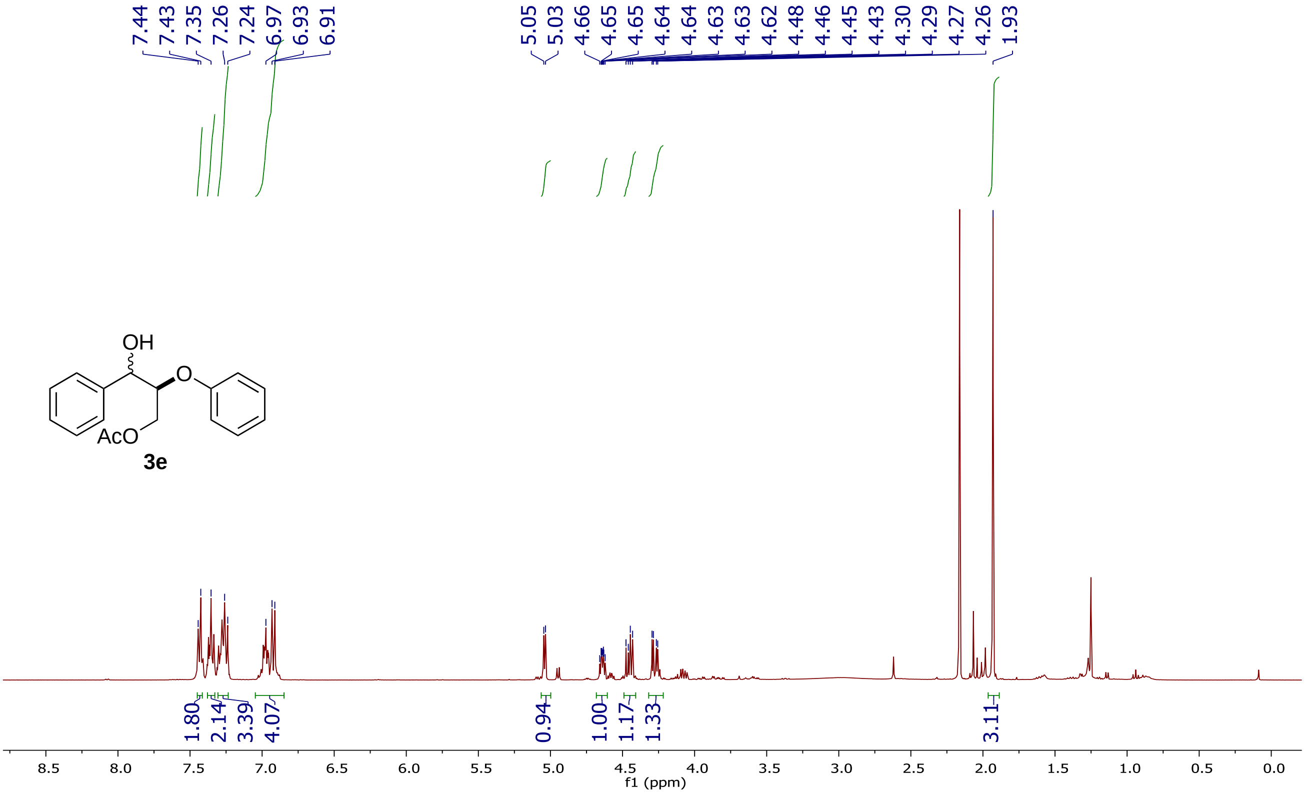

Figure S7: ^{1}H NMR spectrum of **3e.**

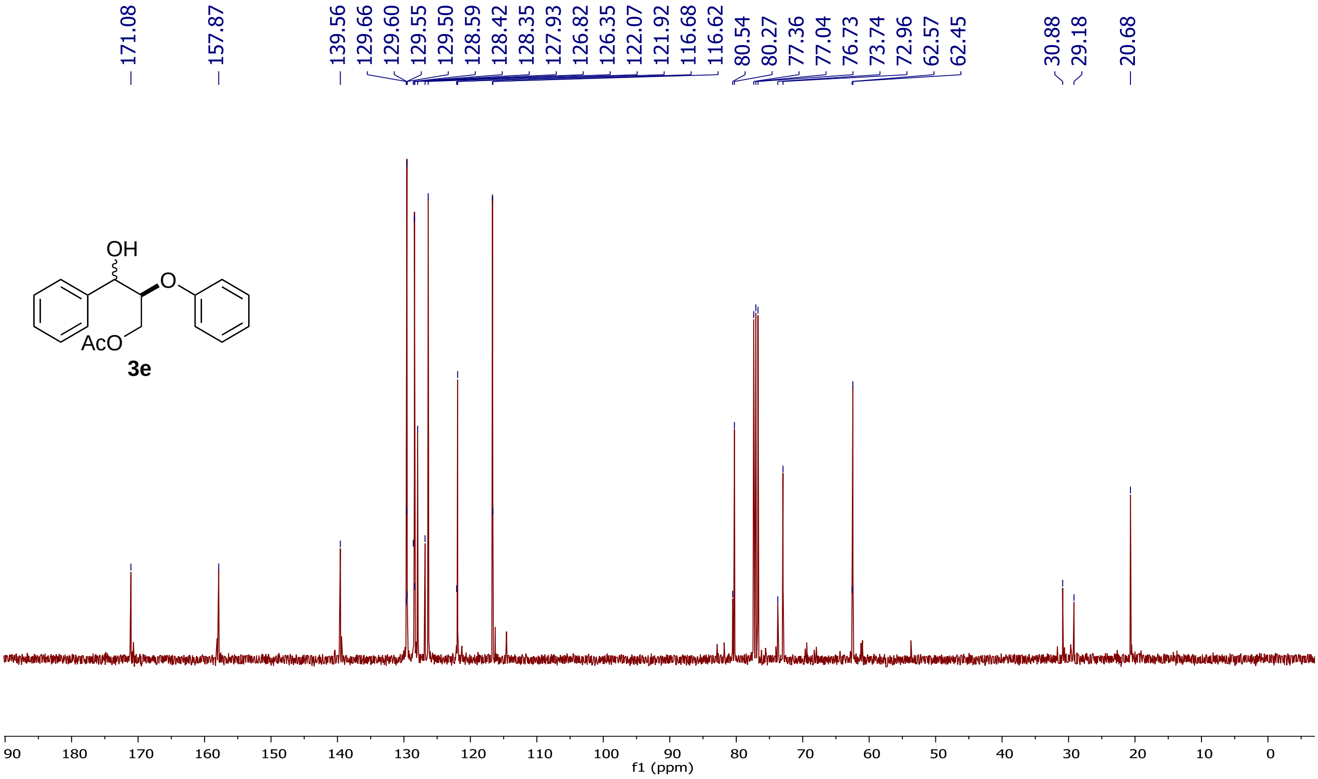

Figure S8: ^{13}C NMR spectrum of **3e.**

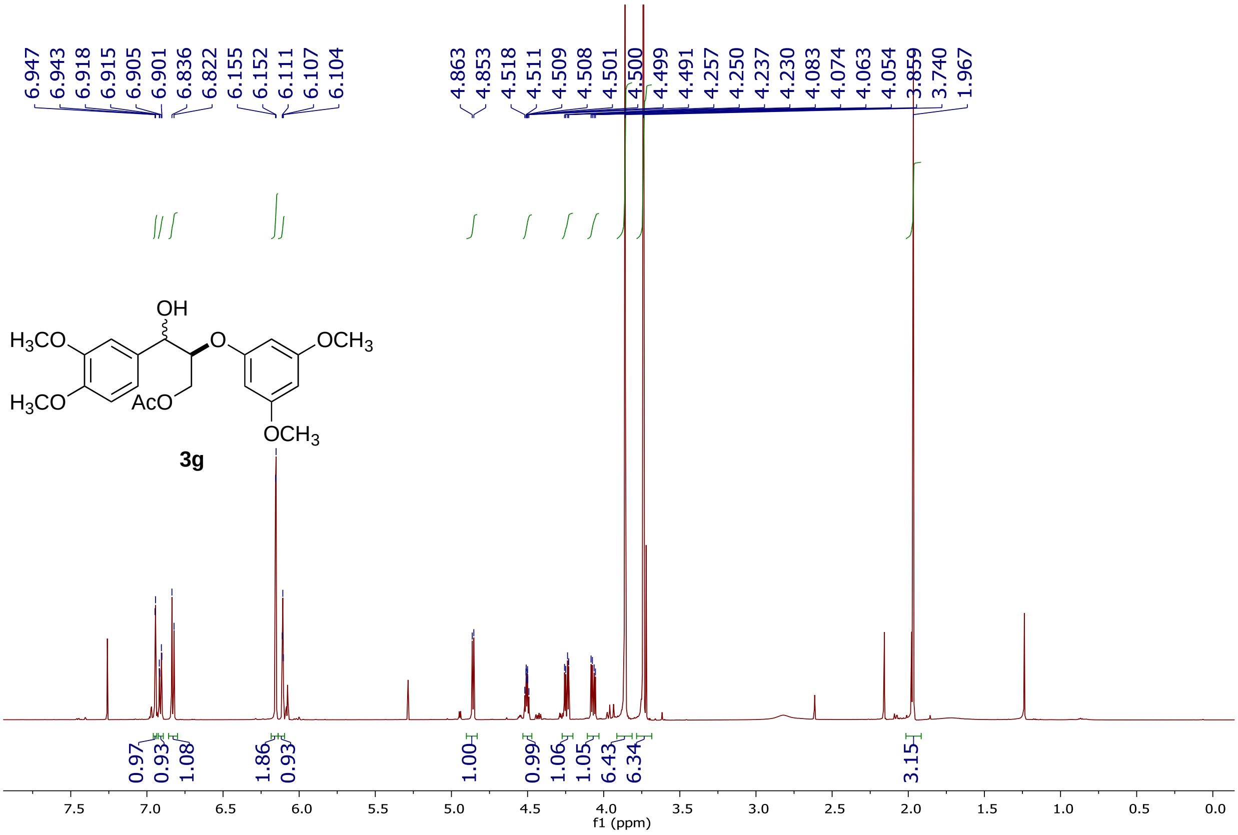

Figure S9: ^{1}H NMR spectrum of **3g.**

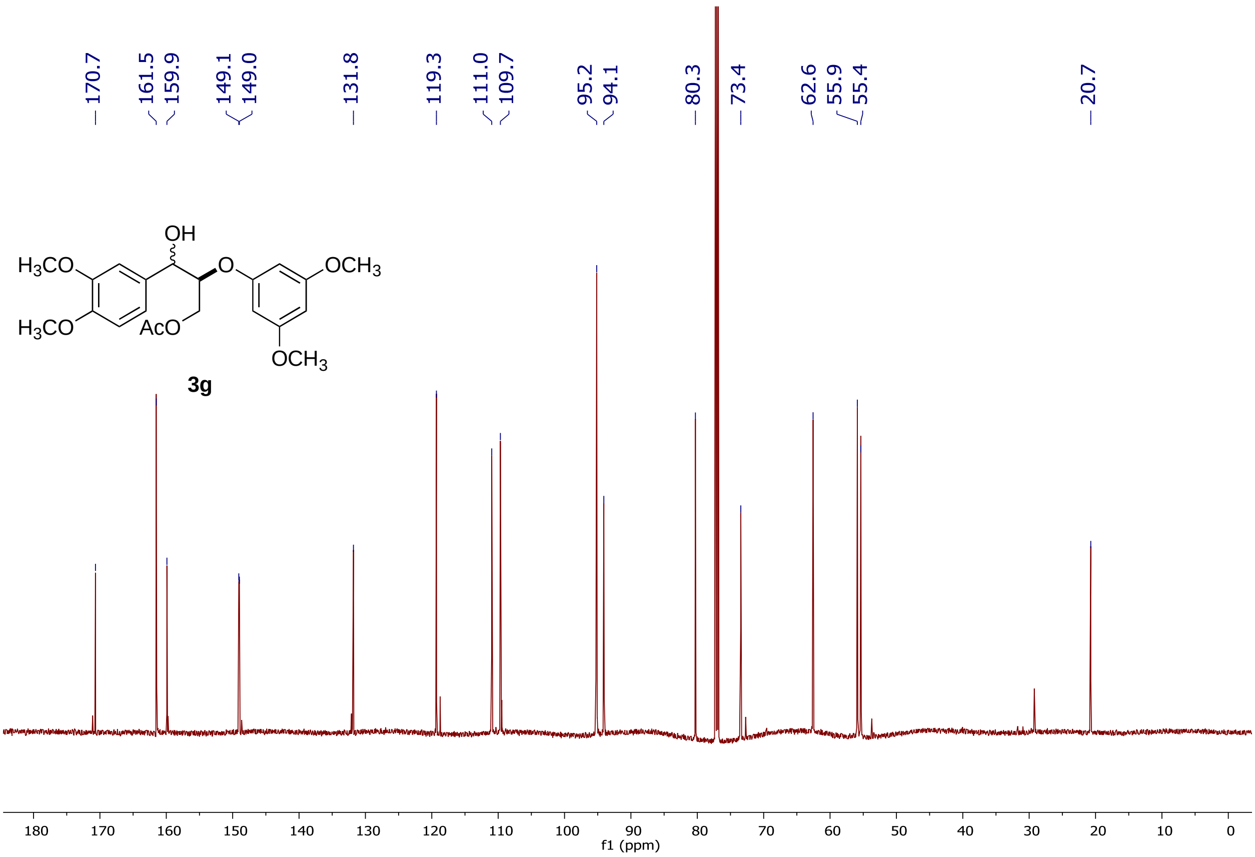

Figure S10: ^{13}C NMR spectrum of **3g.**

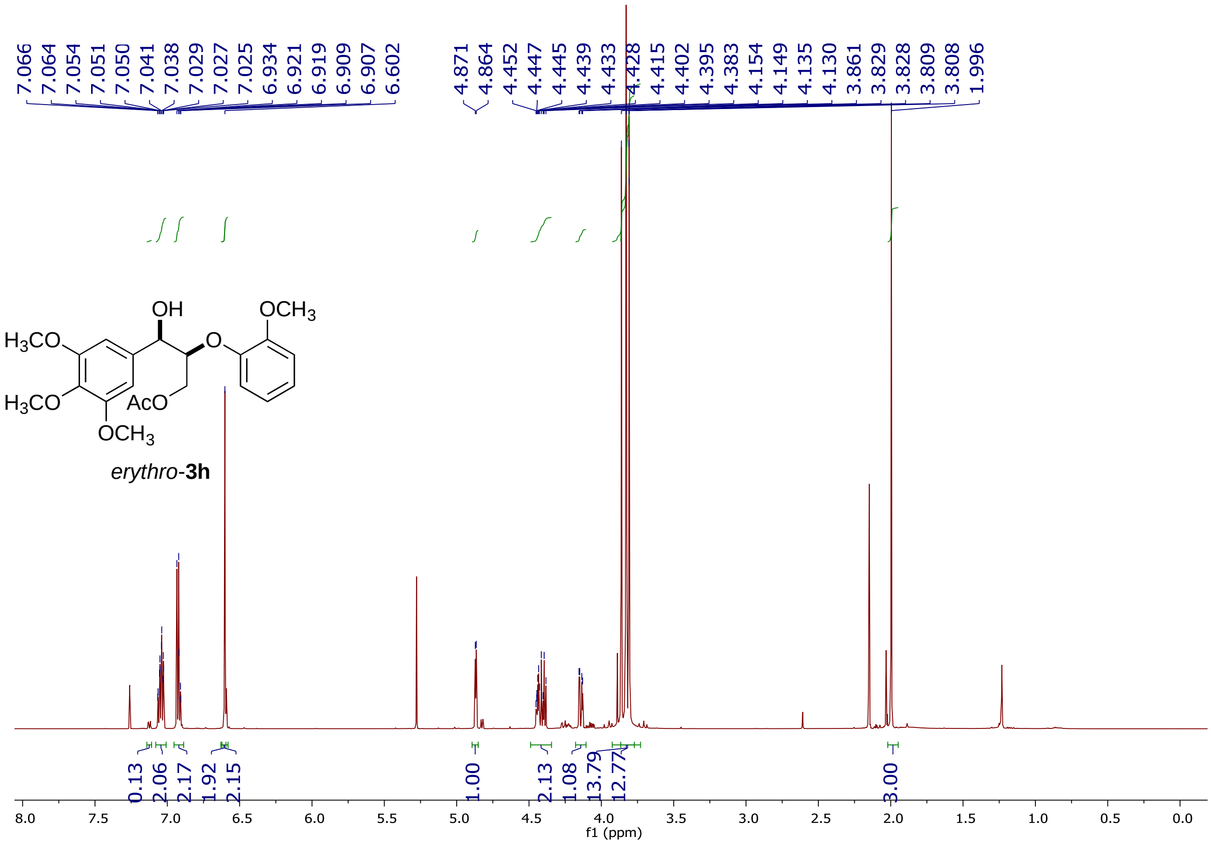

Figure S11: ^{1}H NMR spectrum of *erythro*-**3h.**

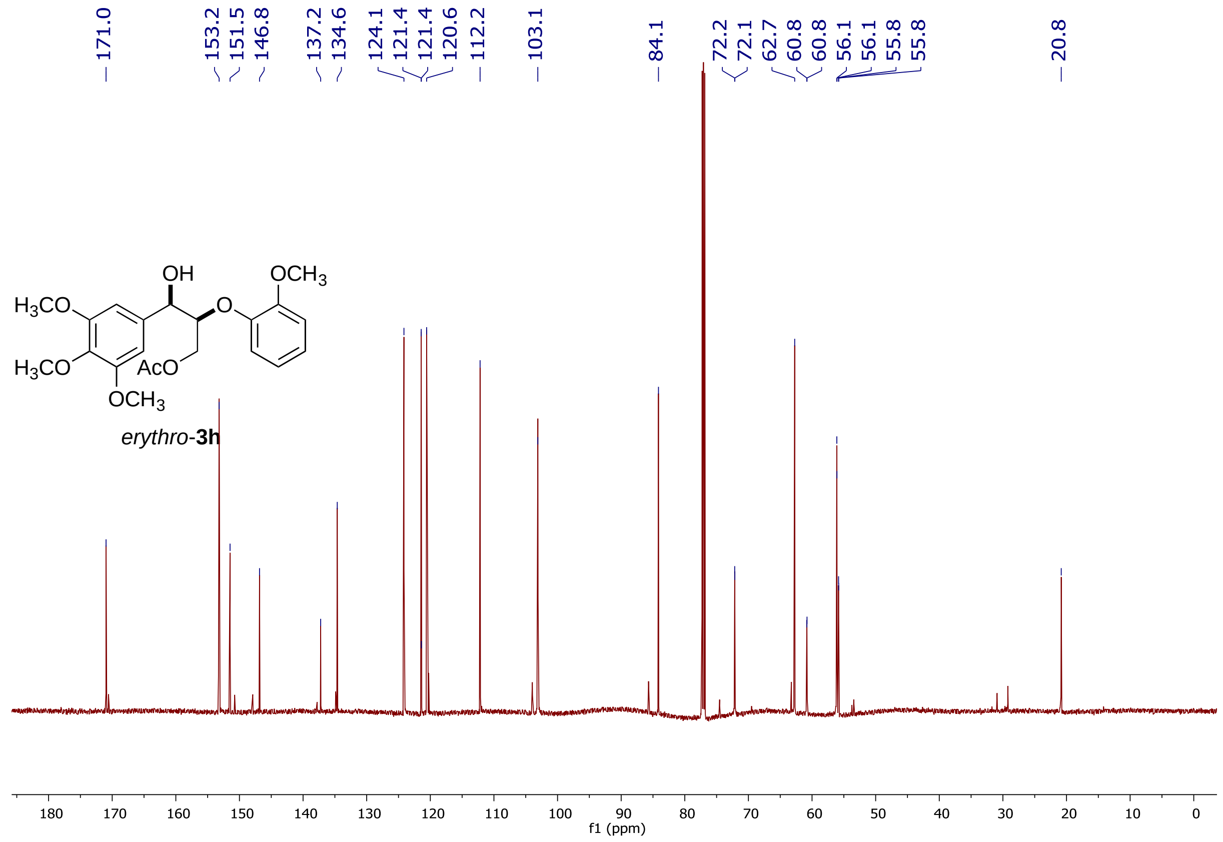

Figure S12: ^{13}C NMR spectrum of *erythro*-**3h.**

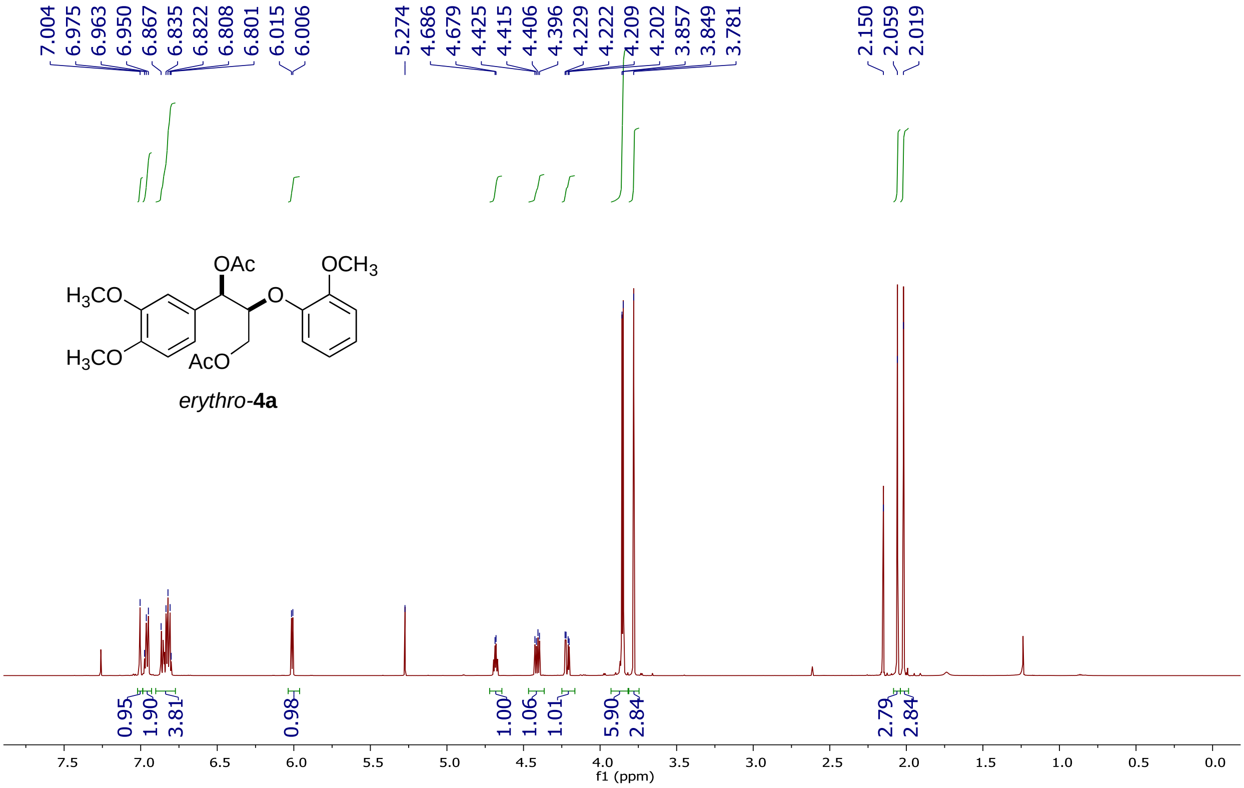

Figure S13: ^{1}H NMR spectrum of *erythro*-**4a.**

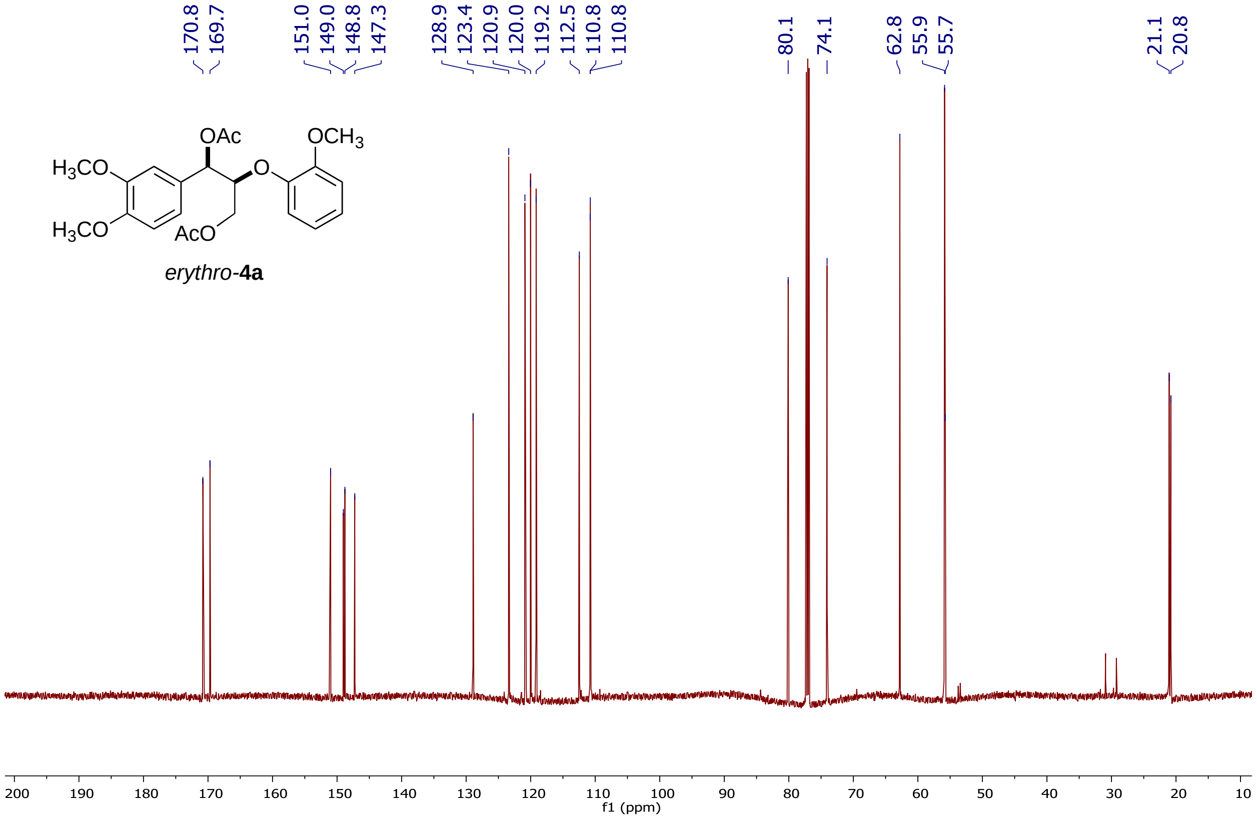

Figure S14: ^{13}C NMR spectrum of *erythro*-**4a.**

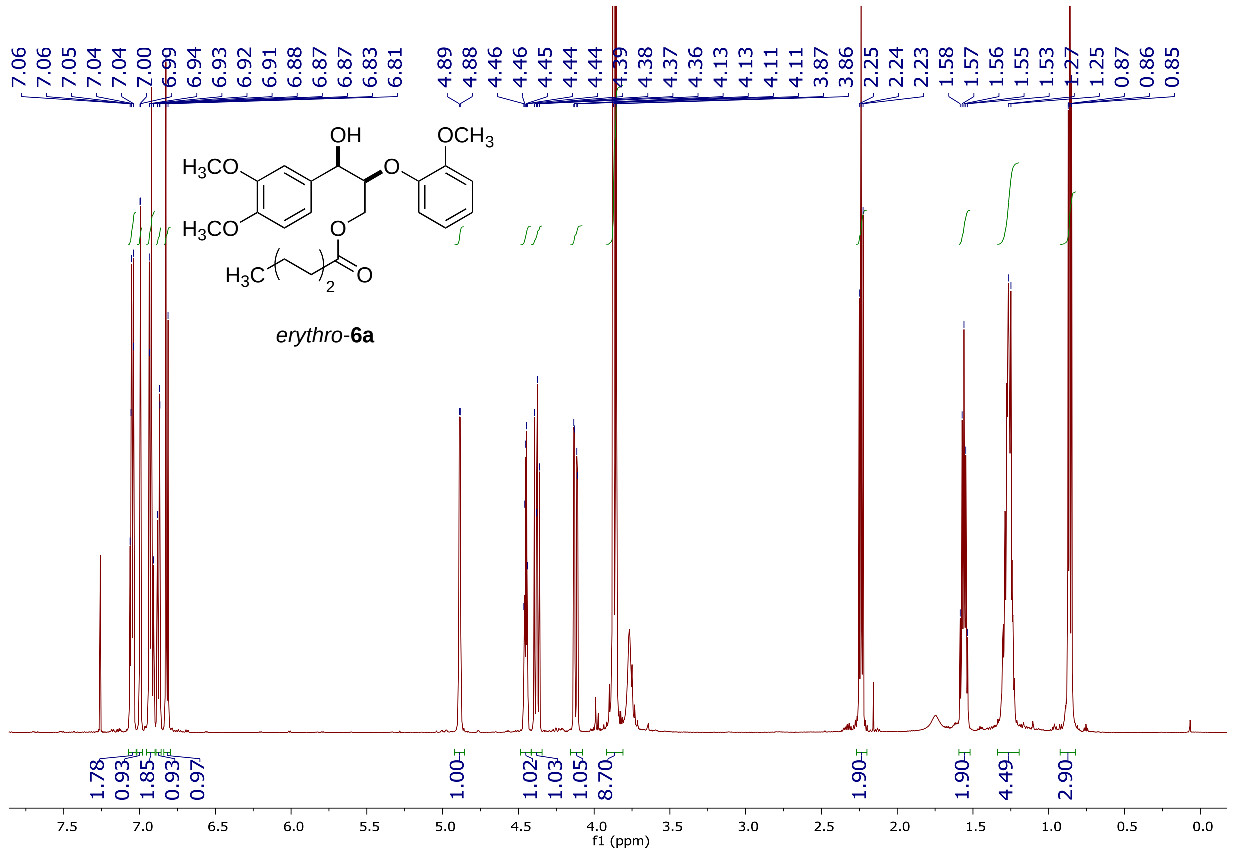

Figure S15: ^{1}H NMR spectrum of *erythro*-**6a.**

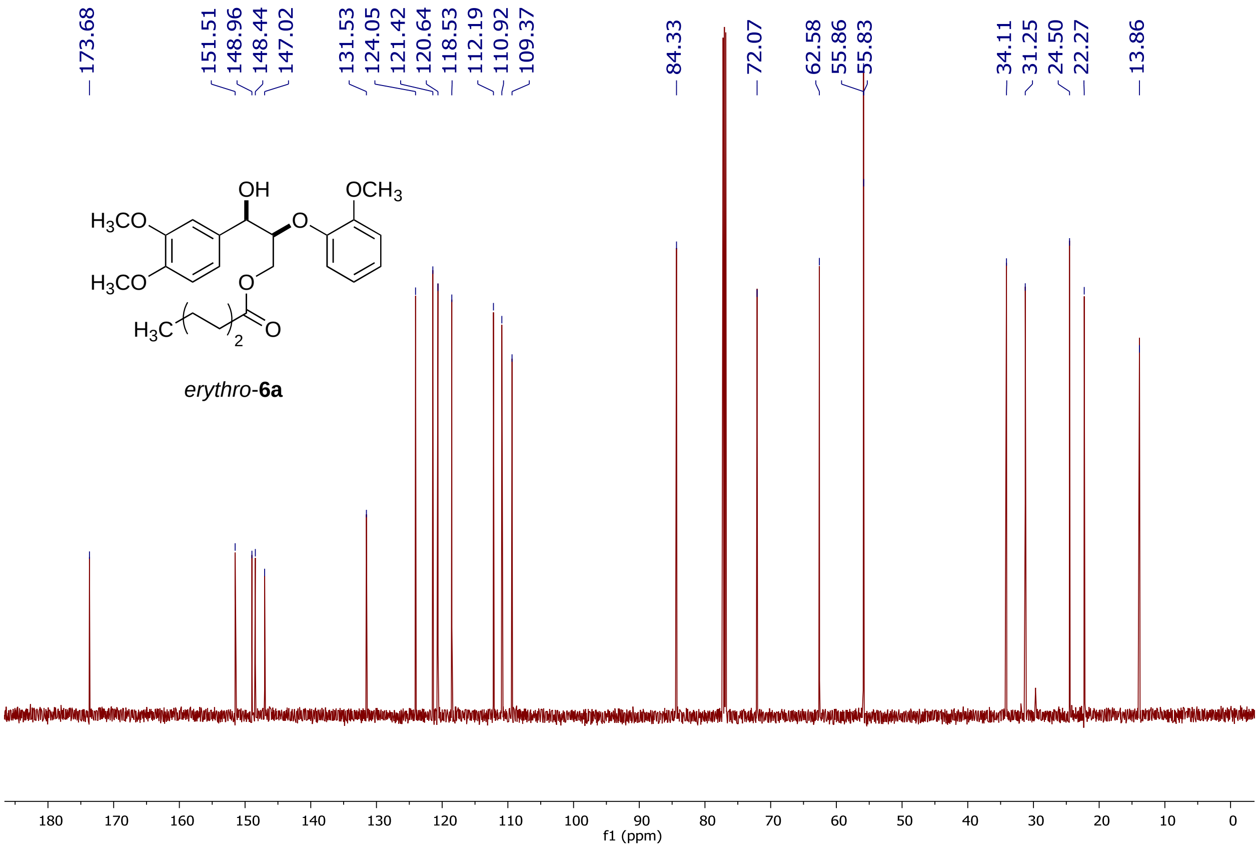

Figure S16: ^{13}C NMR spectrum of *erythro*-**6a.**

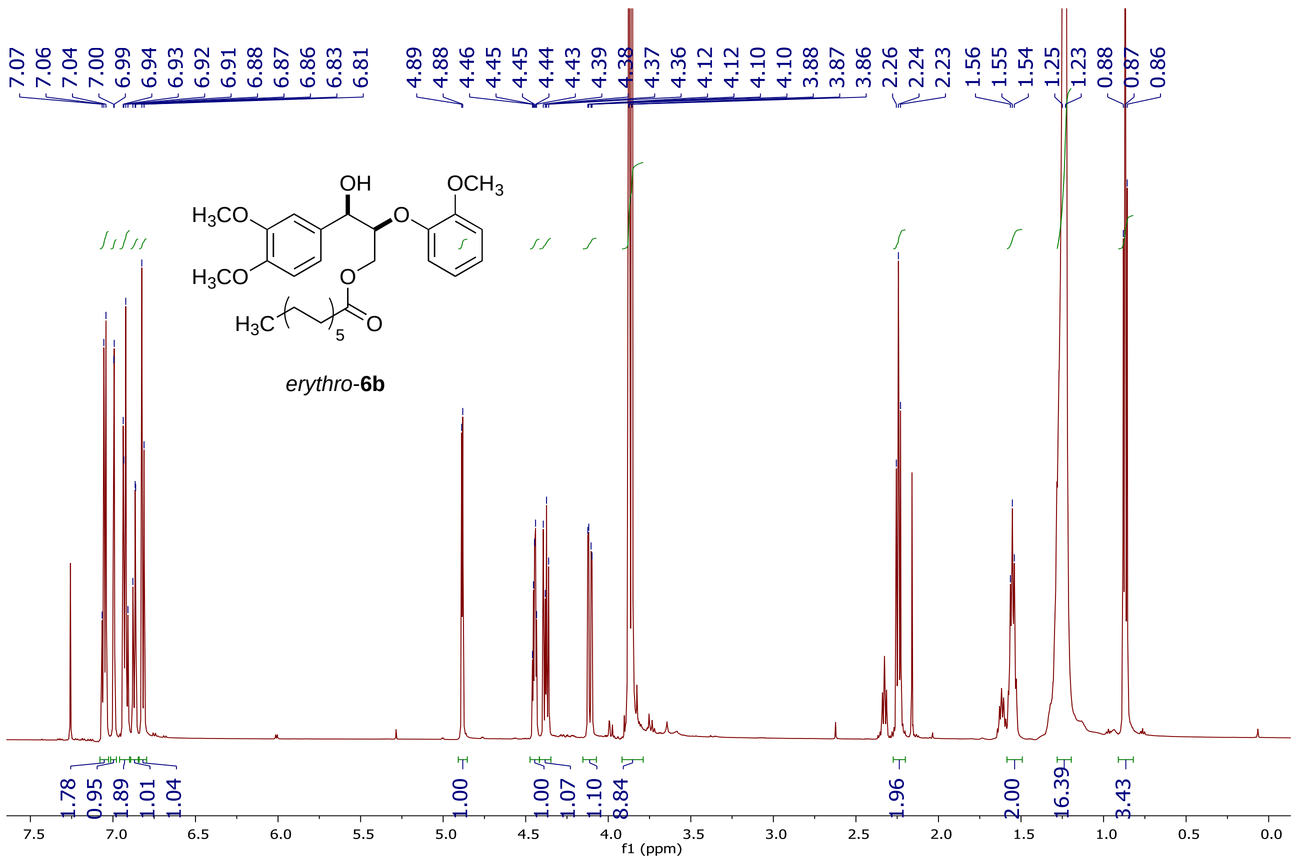

Figure S17: ^{1}H NMR spectrum of *erythro*-**6b.**

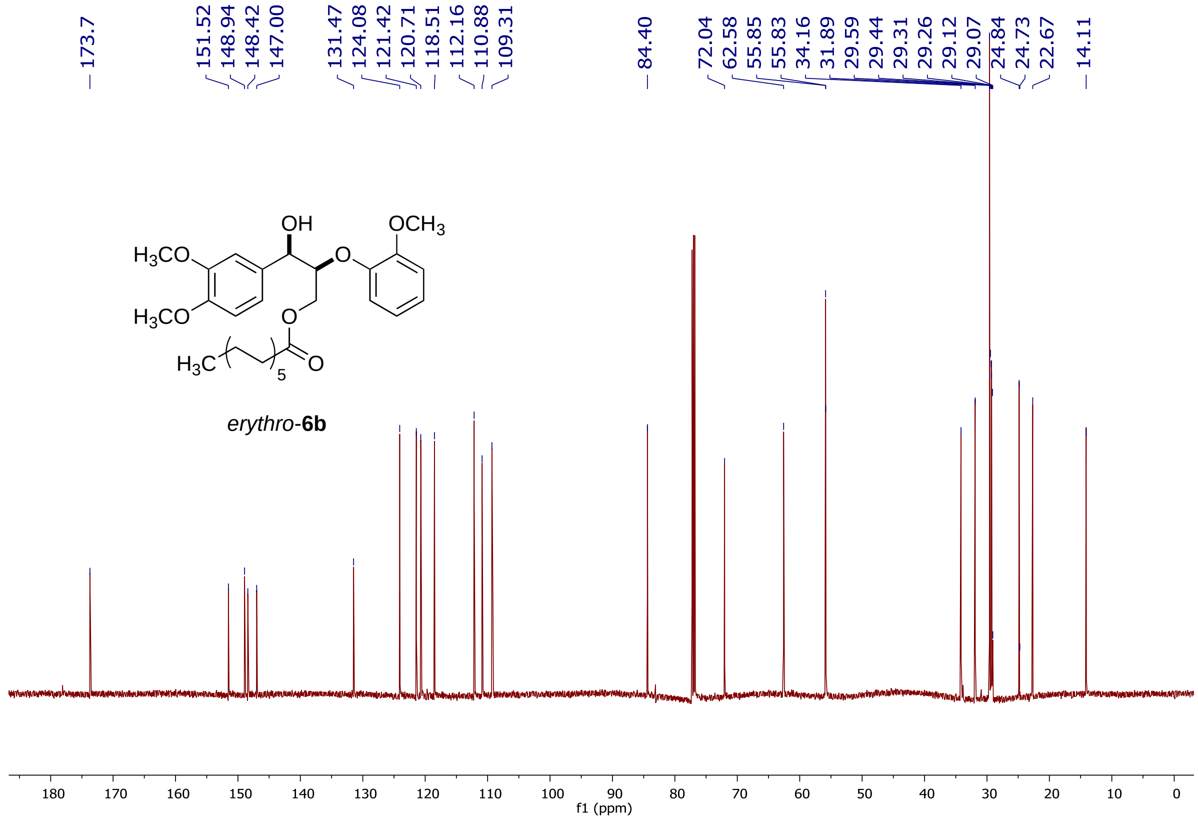

Figure S18: ^{13}C NMR spectrum of *erythro*-**6b.**

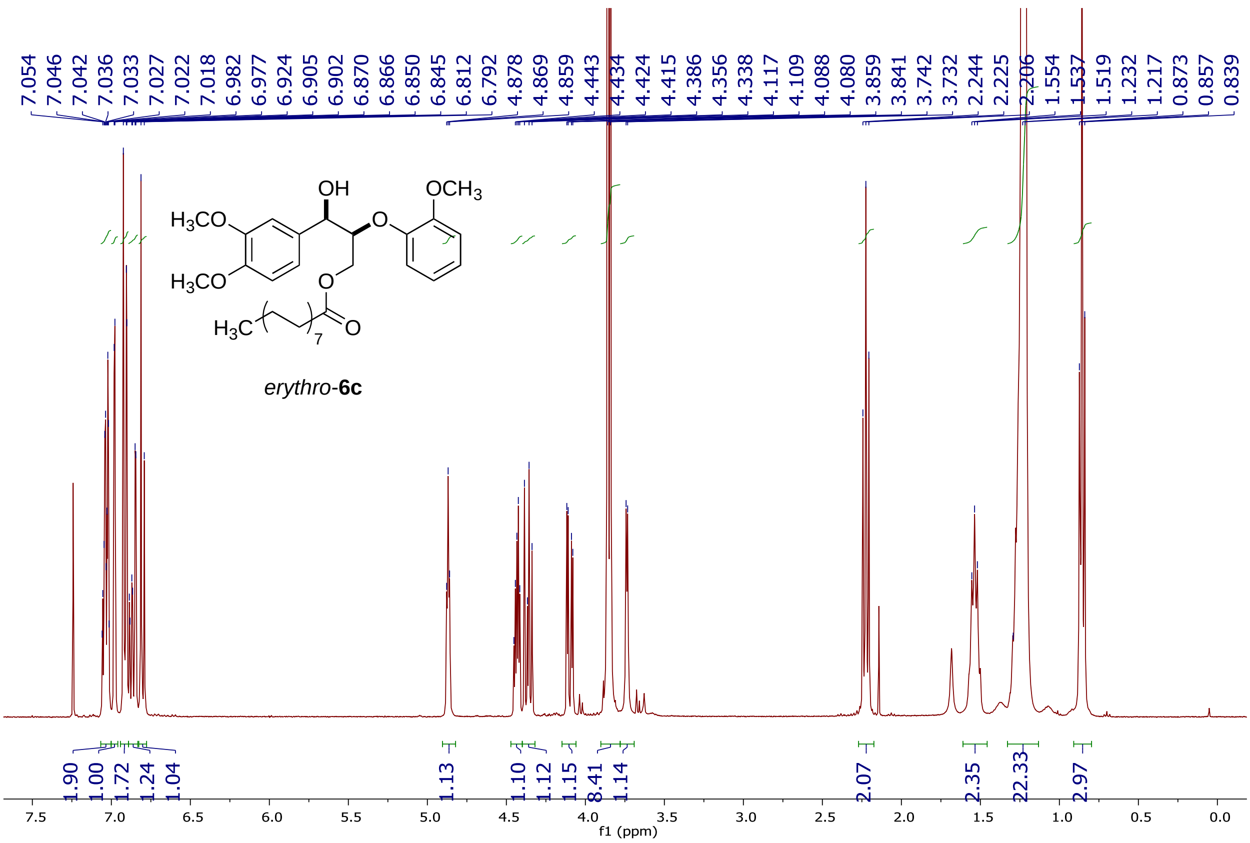

Figure S19: ^{1}H NMR spectrum of *erythro*-**6c.**

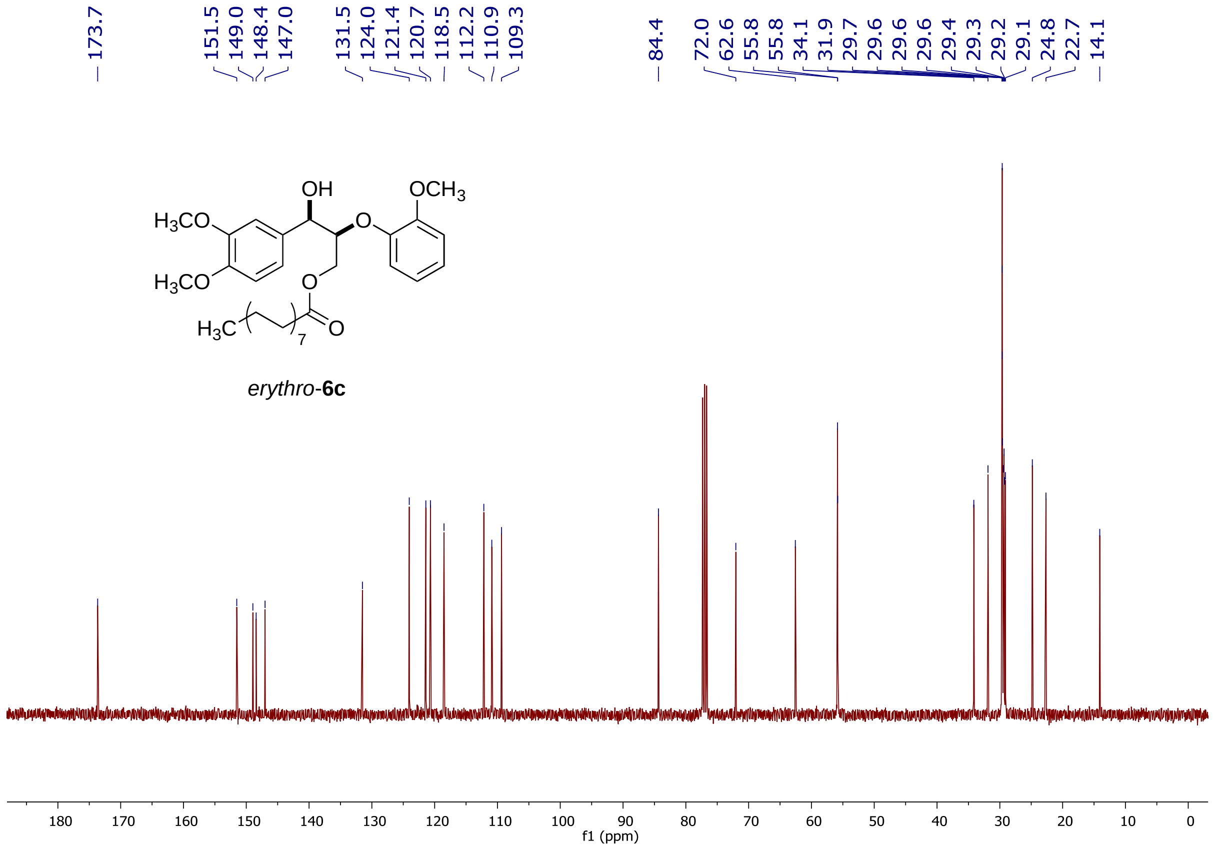

Figure S20: ^{13}C NMR spectrum of *erythro*-**6c.**
